# Electronic Transitions in Different Redox States of Trinuclear 5,6,11,12,17,18‐Hexaazatrinaphthylene‐Bridged Titanium Complexes: Spectroelectrochemistry and Quantum Chemistry

**DOI:** 10.1002/cphc.202000547

**Published:** 2020-10-16

**Authors:** Aleksandra Markovic, Luca Gerhards, Pia Sander, Carsten Dosche, Thorsten Klüner, Rüdiger Beckhaus, Gunther Wittstock

**Affiliations:** ^1^ School of Mathematics and Science Chemistry Department Carl von Ossietzky University of Oldenburg 26111 Oldenburg Germany

**Keywords:** HATN ligand, intervalence charge transfer, quantum chemistry, spectroelectrochemistry, trinuclear Ti complexes

## Abstract

Multinuclear transition metal complexes bridged by ligands with extended π‐electronic systems show a variety of complex electronic transitions and electron transfer reactions. While a systematic understanding of the photochemistry and electrochemistry has been attained for binuclear complexes, much less is known about trinuclear complexes such as hexaphenyl‐5,6,11,12,17,18‐hexaazatrinaphthylene‐tristitanocene [(Cp_2_Ti)_3_HATN(Ph)_6_]. The voltammogram of [(Cp_2_Ti)_3_HATN(Ph)_6_] shows six oxidation and three reduction waves. Solution spectra of [(Cp_2_Ti)_3_HATN(Ph)_6_] and of the electrochemically formed oxidation products show electronic transitions in the UV, visible and the NIR ranges. Density functional theory (DFT) and linear response time‐dependent DFT show that the three formally titanium(II) centers transfer an electron to the HATN ligand in the ground state. The optically excited transitions occur exclusively between ligand‐centered orbitals. The charged titanium centers only provide an electrostatic frame to the extended π‐electronic system. Complete active self‐consistent field (CASSCF) calculation on a structurally simplified model compound, which considers the multi‐reference character imposed by the three titanium centers, can provide an interpretation of the experimentally observed temperature‐dependent magnetic behavior of the different redox states of the title compound in full consistency with the interpretation of the electronic spectra.

## Graphical abstract

**More complex?**! The ground state of the three‐nuclear [(Cp_2_Ti)_3_HATN(Ph)_6_] complex is formed by spin states of different multiplicity and explains the rich electronic transition in the UV‐vis‐NIR ranges in different charge states. Contrary to common notion, IVCT transitions do not play a role in the NIR spectra.

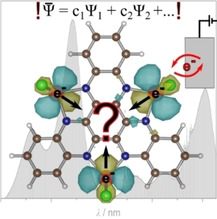

## 1 Introduction

**Figure 1:**
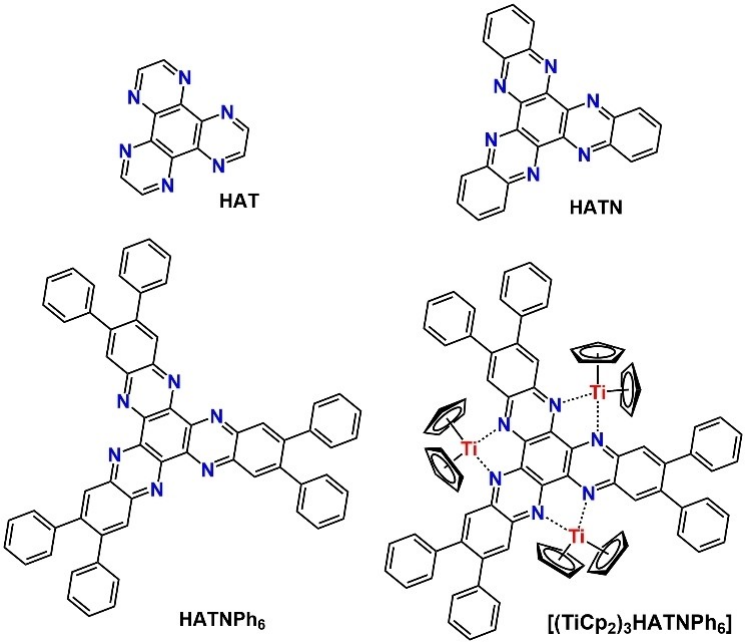
Molecular structure of the ligand HAT, HATN, [HATN(Ph)_6_] and the complex [(Cp_2_Ti)_3_HATN(Ph)_6_].

Electron‐transfer (ET) reactions are fundamental for a lot of processes in nature[^1^, ^2^, ^3^] and numerous investigations have been devoted to the study of ET processes in chemical^[2]^ and biological^[1]^ systems. One of the long‐term goals is the desire to understand ET transfer processes in complex molecular systems that eventually will allow to synthesize molecular systems exhibiting control of electronic communication with similar accuracy as in nature.^[4]^ The work of Creutz and Taube^[5]^ on mixed‐valence compounds already illustrated the complexity of processes at binuclear complexes that bear conceptual similarity to ET reactions in natural systems.^[3]^ In particular, polypyridyl complexes of the d^6^ metals Fe(II), Ru(II), and Os(II) have received considerable attention due to their chemical inertness in a variety of oxidation states.^[3]^ While a systematic understanding of the photochemistry and electrochemistry has been attained for binuclear complexes,^[6]^ much less is known about trinuclear and multinuclear complexes.^[7]^ Trinuclear complexes can be obtained by using hexaazatriphenylene (HAT),^[8]^ hexaazatrinaphthylene (HATN) ligands^[9]^ and backbone substituted derivatives^[10]^ thereof. Here we present investigations of the hexaphenyl substituted isomer (HATN (Ph)_6_) and the corresponding titanocene complex (Figure 1).[^11^, ^12^]

In extension of the classical concepts of the coordination chemistry, multinuclear derivatives exhibiting bridging *N*‐heterocyclic ligands are discussed by Haiduc as inverse coordination metal complexes.^[13]^ Trinuclear complexes of early transition metals, are particular interesting if they contain bridging ligands with extended π‐acceptor properties. (Figure 1).^[10]^ Complexes of this type have been extensively studied as attractive supramolecular building blocks because those ligands possess three chelating sites for metal ions.^[10]^ Secondly, the electron‐deficient π‐system of the ligand supports metal‐to‐ligand charge transfer and/or charge transfer between metal centers of the complex.^[10]^ Thirdly, the C_3_ symmetry axis causes degeneracy of π* orbitals.^[10]^ Fourthly, the ligands offer a variety of interesting chemical/physical properties based on their electronic structures which can be modified by their peripheral substituents.^[10]^

HAT and its derivatives also belong to the family of redox‐active ligands, often used as non‐innocent ligands.^[14]^ One of the fundamental properties of these ligands is their ability to act as electron reservoirs, which allows the metal to store electrons on the ligand and/or accept electrons from the ligands.^[15]^ This property is of interest for the design of homogeneous catalysts because many important transformations involve the transfer of multiple electrons between the catalyst and the activated substrate. Such transformations are common for expensive noble metals such as Pd, Pt, Rh, etc., but more difficult to achieve with cheaper and more abundant first‐row transition metals. Non‐innocent ligands actually allow first‐row transition metals to mimic some of the catalytic properties of more noble metals.^[16]^ In addition, the electron transport properties of complexes with non‐innocent ligands have been studied for applications in redox‐flow batteries.^[17]^

Because of all the characteristic multifunctionalities of HAT‐type ligands, their metal complexes afford intriguing solid state structures, physicochemical and material properties and have opened up a new field of trinuclear coordination compounds.^[10]^ Complexes with ruthenium,^[18]^ rhenium,^[19]^ cobalt^[14]^ and titanium^[7]^ have been investigated concerning their interesting electrochemical, photophysical and magnetic properties. Among others, the frequent occurrence of mixed‐valence situations have been emphasized.[^20^, ^21^, ^22^] In the seminal review of Kaim and Lahiri,^[20]^ the intervalence charge Transfer (IVCT) is related to spectral features: „Frequently, the intervalence charge‐transfer absorption band have been recognized as the most conspicuous evidence for a mixed valence situation. They arise from intramolecular electronic transitions…“ and “The IVCT band is usually observed in the visible or near infrared region of the spectrum and is broad.” This notion is commonly accepted and even popularized in Wikipedia.^[23]^ The extend of coupling between redox centers is commonly summarized by the three Robin‐Day classes for negligible (I), weak (II) and strong (III) coupling of the redox centers.[^21^, ^24^] Spectroscopic methods are recommended for the analysis of those situations.^[21]^

In this study, we investigate the electronic transitions in the different redox states of the trinuclear titanium hexaphenyl‐5,6,11,12,17,18‐hexaazanaphtylene. (Figure 1).^[12]^ The results have been obtained by extended voltammetric measurements and spectroelectrochemistry in the ultraviolet (UV), visible (vis) and near infrared (NIR) spectral regions. The initial, preliminary assessment of the NIR spectra as signatures of intramolecular electronic transitions[^7^, ^12^] with IVCT, ligand‐to‐metal charge transfer (LMCT) and metal‐to‐ligand (MLCT) processes could not provide a contradiction‐free assignment of all spectral features despite its gross agreement with common textbook knowledge about mixed‐valence compounds and their electronic spectra.^[23]^ This became only evident when considering the spectra of the different redox forms accessible by chemical synthesis or by oxidation/reduction in a spectroelectrochemical cell. Density functional theory (DFT) using linear response time‐dependent DFT and complete active space self‐consistent field (CASSCF) provided a surprising, alternative and comprehensive interpretation of the electronic structure, temperature‐dependent magnetic behavior and the resulting electron transition. Those methods consider true multi‐electron transitions rather than the single electron picture underlying the Robin‐Day classification and its extensions.

## 2 Results and Discussion

### 2.1 Electrochemistry of [(Cp_2_Ti)_3_HATN(Ph)_6_] and the Ligand HATN(Ph)_6_

**Figure 2:**
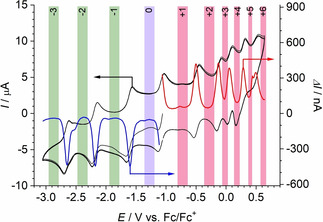
CV (black) and DPV (red, blue) of 0.3 mM [(Cp_2_Ti)_3_HATN(Ph)_6_] at an Au working electrode in 0.2 M TBAPF_6_ in THF. The shaded areas indicate in which potential range the complex is stable with the indicated total charge.

The cyclic voltammetry (CV) and differential pulse voltammetry (DPV) data of [(Cp_2_Ti)_3_HATN(Ph)_6_] in THF are shown in Figure 2. DPV were started at the open circuit potential (OCP) in the positive direction in order to record the oxidations (red) and in the negative direction in order to record the reductions (blue). The potential range, in which the neutral complex was stable, is colored in lavender, the potential range of the negatively charged species are marked in green and potential ranges in which the positively charged species are stable are marked in red. The DPV data are overlaid with the CV data because DPV data show a better resolution of the redox processes on the potential axis. Please note the separate ordinates for both CV and DPV experiments.

The voltammogram of [(Cp_2_Ti)_3_HATN(Ph)_6_] shows three reduction waves and six oxidation waves. All steps are one electron processes (Table 1). The six oxidations formally correspond to the removal of 6 valence electrons of the three Ti(II) centers. The rich redox chemistry of this [(Cp_2_Ti)_3_HATN(Ph)_6_] allows the observation of even one more redox transition in the accessible potential range than for the previously reported related compound^[7]^ where 3 reductions and 5 oxidation signals were observed

**Table 1:** Formal potentials (in V vs. Fc/Fc^+^) for electron transfer reactions of [(Cp_2_Ti)_3_HATN(Ph)_6_] from the data in Figure 2.

Redox pair	*E*°’
−3/−2	−2.66
−2/−1	−2.19
−1/0	−1.61
0/1	−1.08
1/2	−0.49
2/3	−0.12
3/4	+0.06
4/5	+0.24
5/6	+0.49

**Figure 3:**
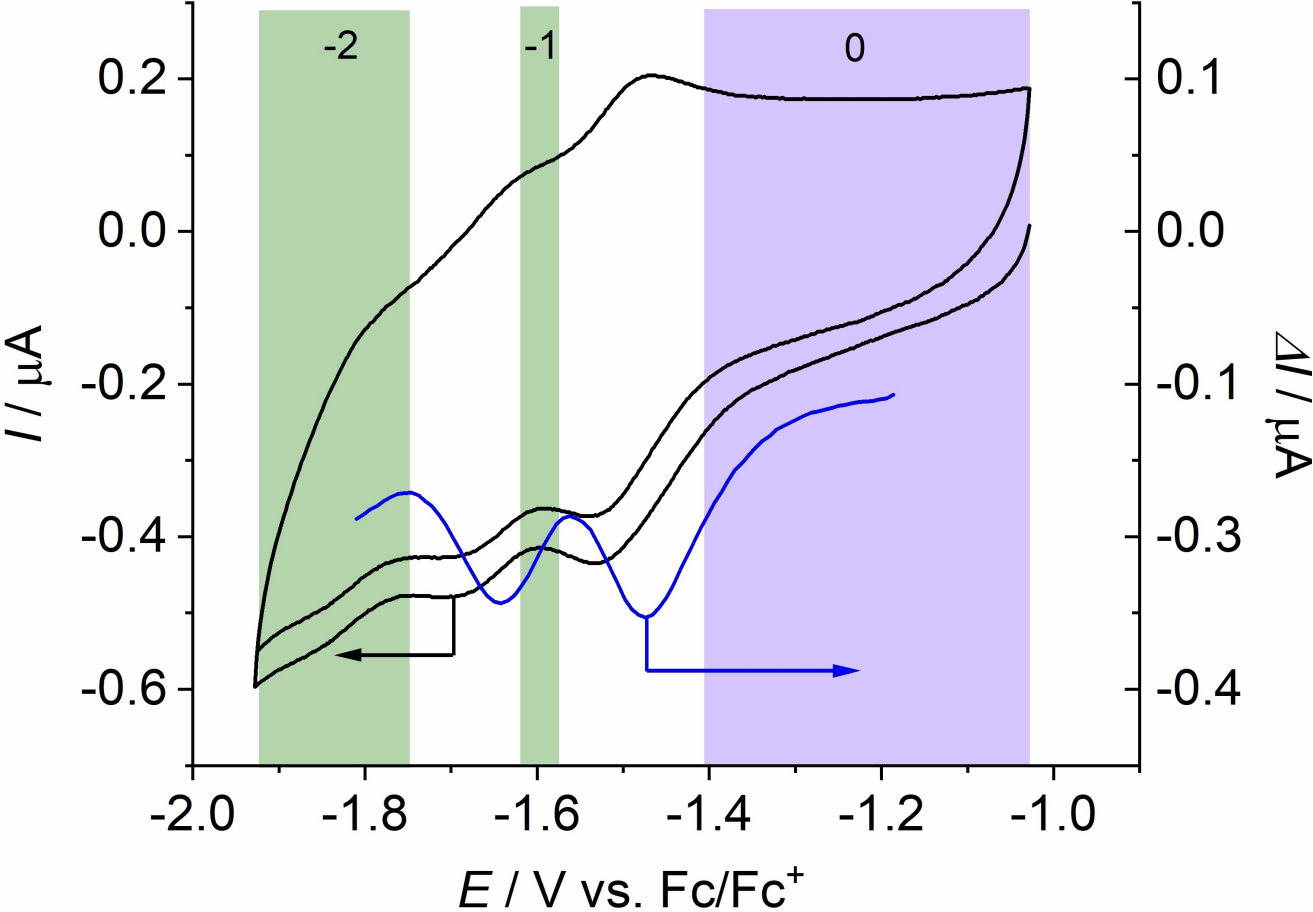
CV (black) and DPV (blue) of 0.1 mM HATN(Ph)_6_ (no metal ions coordinated) at an Au disc working electrode in 0.2 M TBAPF_6_ in a mixture of 50 vol‐% THF and 50 vol‐% MeCN. The shaded areas indicate in which potential range the molecule is stable with the number of accepted electrons compared to the neutral starting state.

By comparison of the voltammetric data of the complex (Figure 2) with that of the ligand HATN(Ph)_6_ (Figure 3), the reductions 0→−1 and −1→−2 suggest that they lead to reduction of the ligand. However, such assignment based on voltammetric data alone can never be certain. The third reduction of the complex and −2→−3 cannot associated with a redox transition of the ligand alone.

### 2.2 Spectroelectrochemical Measurements

**Figure 4:**
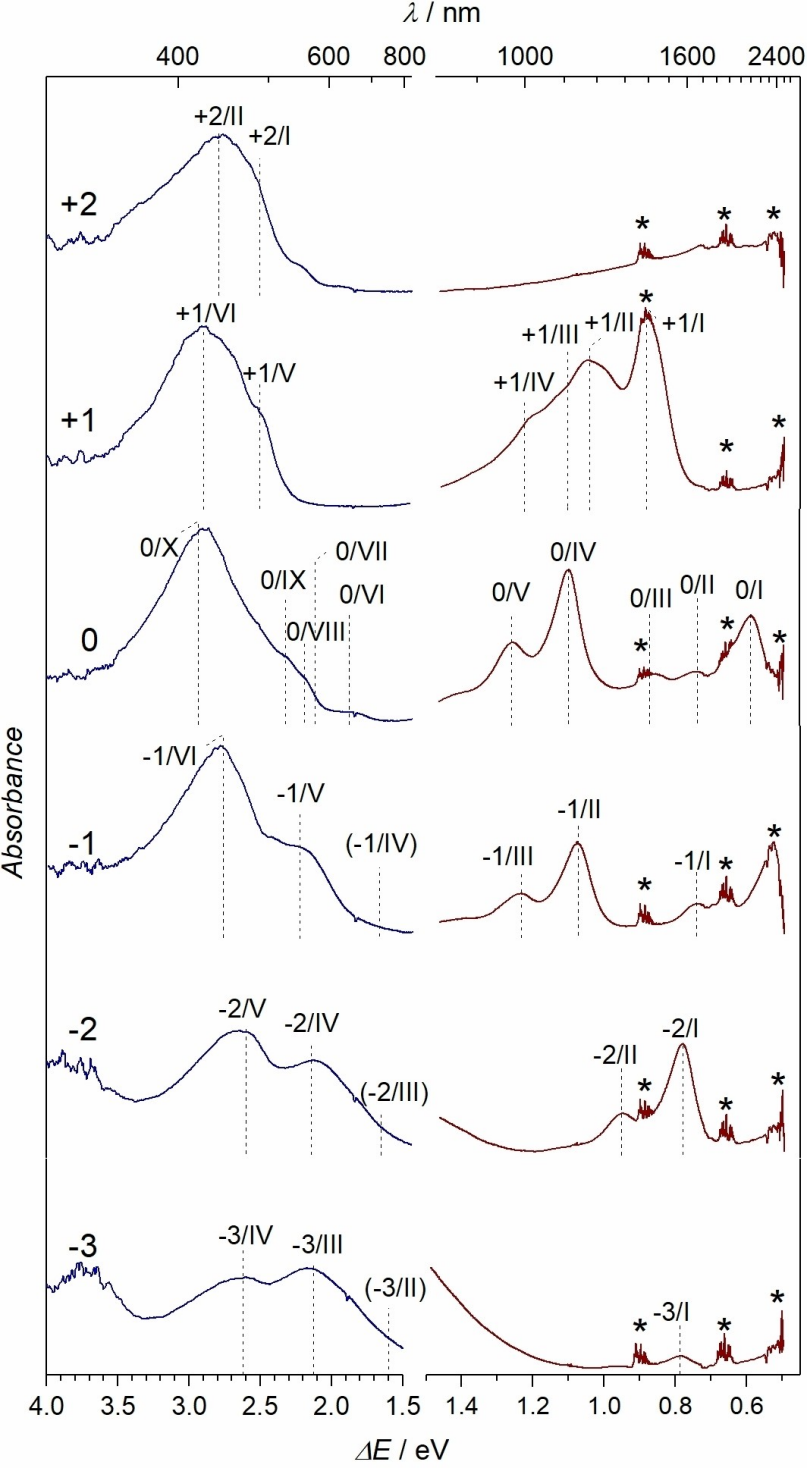
The spectra from the spectroelectrochemical measurements of [(Cp_2_Ti)3HATN(Ph)_6_] in THF; UV/Vis in blue and NIR in red. The signals are numbered for later reference. Signals marked by (*) are caused by vibrational overtone of molecular vibration of the solvent, which are not fully compensated. They are not relevant for the interpretation of the electronic transitions.

The solution spectra of [(Cp_2_Ti)_3_HATN(Ph)_6_] and its oxidation and reduction products [(Cp_2_Ti)_3_HATN(Ph)_6_]^3−^, [(Cp_2_Ti)_3_HATN(Ph)_6_]^2−^, [(Cp_2_Ti)_3_HATN(Ph)_6_]^1−^, [(Cp_2_Ti)_3_HATN(Ph)_6_]^1+^ and [(Cp_2_Ti)_3_HATN(Ph)_6_]^2+^ show electronic transitions in the UV, vis and NIR spectral ranges (Figure 4).

**Figure 5:**
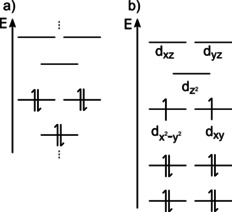
Schematic MO‐diagrams for a) HATN (no metals coordinated) and b) Cp_2_Ti. HATN has two degenerate HOMOs due to its symmetry (orbital shape is shown in SI‐3, Figure S4). The Cp_2_Ti complex has two unpaired electrons localized in the d_x_^2^_‐y_^2^ and d_xy_ orbitals. For lower lying orbitals a strong mixing between cyclopentadiene and titanium is observed. All calculations were made using PBE0 and Def2‐SVP.

Different types of electronic transitions can be observed in transition metal complexes. In common literature they are usually visualized by molecular orbital (MO) diagrams, where MOs are combined from ligand orbitals (Figure 5a) and metal d orbitals in a ligand field of the Cp ligands (Figure 5b).[^25^, ^26^] Electronic states in this approach are represented as linear combinations of the occupied MOs. In a single electron picture, transitions between these states can be included in the MO scheme as electron transitions between distinct orbitals.^[26]^ Possible transitions are transitions from ligand‐centered orbitals to d‐type metal orbitals (LMCT), transitions between d‐type orbitals within *one* metal center (d‐d transitions), transitions from metal centered d‐type orbitals to ligand orbitals (MLCT) and of course transitions between purely ligand centered orbitals (in the visible region mainly π‐π*). In multinuclear complexes transitions between d‐type orbitals of different metal centers in different valence states can be observed as a fifth type of electronic transition, commonly referred to as IVCT. However, an IVCT is only possible in systems with extensive state mixing involving two d orbitals from the metal centers and one ligand orbital which provides strong electronic coupling between the metal centers.^[20]^

Experimental magnetic moments of the solid compound at elevated temperature suggest the existence of more than two unpaired electrons in the ground state of the neutral complex.^[12]^ Due to the high electron affinity of the HATN(Ph)_6_ ligand at least one electron is supposed to be transferred from the three Cp_2_Ti centers to the ligand. The cyclic voltammetric data of HATN(Ph)_6_ in Figure 3 showed that the two‐electron reduction was irreversible suggesting that the [HATN(Ph)_6_]^2−^ was unstable in the electrolyte solution. The combined magnetic and electrochemical data made the ground state plausible with a negatively charged ligand, although the exact number of transferred electrons cannot be ascertained with the available methods.

The measured spectra displayed in Figure 4 exhibit pronounced peaks within the NIR range which in many publications are assigned as an IVCT or LMCT.[^20^, ^21^, ^22^] For an assignment of these peaks and to combine the spectroscopic data with the electrochemical data, we attempted to relate the formal potential of electron transfer reactions (Figure 2, Table 1) and all spectroscopic transitions to an assumed single particle MO scheme for all redox states. From the already published magnetic data, the transfer of one electron to the HATN in the ground state was assumed. This attempt is fully documented and its severe limitations are discussed in the Supporting Information (SI‐2).

While this assignment seemed plausible at first sight, it can *not* provide a full explanation for the spectra of the oxidized species, i. e. there is disappearance of the expected CT bands in experimental NIR spectra. For the HATN complexes, this is not surprising because the applicability of the single electron picture and the appropriate definition of the ground state require more detailed quantum chemical calculation as provided in Sections 2.3. to 2.5. of this paper. For [(Cp_2_Ti)_3_HATN(Ph)_6_] studied here, one must suspect appearance of multiple spin states. Those scenarios prevent a meaningful applicability of a single electron picture in addition to the anyway very crude assumptions required to condense electrochemical and spectroscopic data in one picture. Therefore, we strongly discourage to derive theoretical quantities such as orbital energies from experimental data in case high quality quantum chemical calculations are available.

### 2.3 Calculation of the Electronic Ground State and Electronic Excitations

In order to understand the ground state configuration and electronic excitations, the electronic structure of the HATN molecule must first be examined more closely without coordinated metal ions. Due to its D_3h_ symmetry two degenerate HOMOs can be found (Figure 5a). In the literature, a large electron affinity of the HATN ligand is reported.^[14]^ This can also be shown in first approximation by a PBE0/Def2‐SVP/RIJCOSX calculation of the charged species (−1, −2 and −3. From those data, the electron affinities *E*_af_ in Table 2 were obtained by [Eq. (1)]:

(1) $$E{}_{\mathrm{Af}}=E{}_{\mathrm{charged}}\text{-}E{}_{\mathrm{neutral}},$$

**Table 2:** Electron affinities of HATN (no metals coordinated) in different charge states. The energetically lowest lying spin state with no spin contamination were used for comparison.

Charge *Q*|Spin *S*	Energy [a.u.]	*E*_af_ [eV]
0|0	−1248.008	0.00
−1|1/2	−1248.070	−1.69
−2|0	−1248.014	−0.15
−3|3/2	−1247.836	4.69

where *E*_charged_ and *E*_neutral_ represent the energies of the charged species and of the neutral molecule, respectively.

Each Cp_2_Ti has up to two unpaired electrons that can be transferred while bonding with the HATN molecule (Figure 5b). The electronic structure of Cp_2_Ti complexes is detailed investigated in literature.^[27]^ The data in Table 2 and the fact that Cp_2_Ti is known as a strongly reducing agent make it plausible that a charge transfer from Cp_2_Ti to the HATN ligand is present in the electronic ground state of [(Cp_2_Ti)_3_HATN(Ph)_6_]. Generally, the bent titanocene(II) fragment is often used in the preparative chemistry in order to introduce electron transfer processes as starting point for a broad range of subsequent reactions.^[28]^ In mononuclear 2,2‐bipyridine titanocene complexes, the chelating ligand is well known as redox active.^[29]^ According to our calculations up to three electrons might be transferred. Although, the transfer of three electrons to the isolated ligand is energetically disfavored, stabilization by Coulomb forces from Cp_2_Ti leaves this transfer as a valid option.

**Figure 6:**
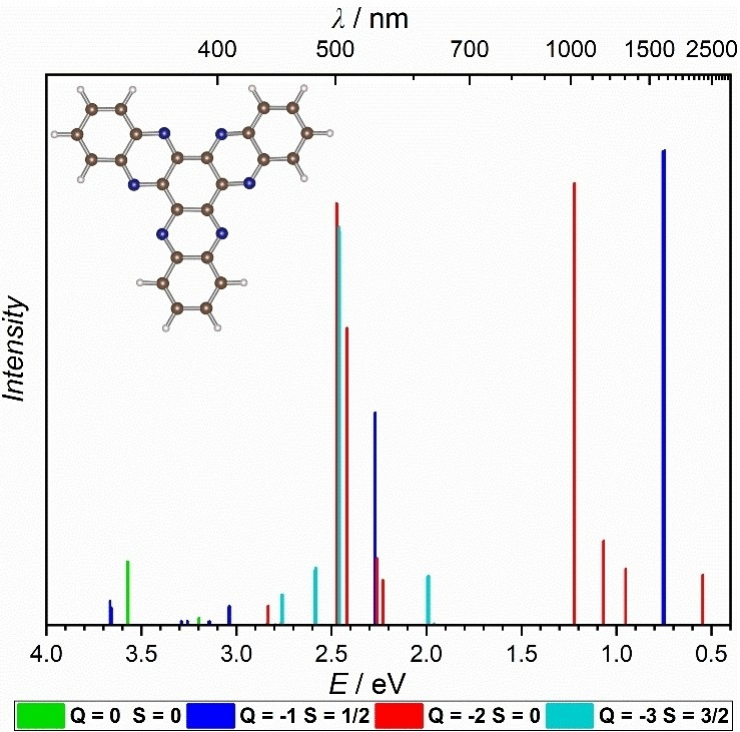
Calculated absorption spectra of HATN ligand on TD‐PBE0/Def2‐SVP level with different charges (Q) and spin (S).

Calculation of spectra of the charged HATN molecule with no metal coordinated within time‐dependent density functional theory (TD‐DFT) give first insights into the excitations observed in the experimental NIR spectra. When the HATN molecule is charged, the calculation reproduces similar electronic excitations as found for the [(Cp_2_Ti)_3_HATN(Ph)_6_] in the NIR range (Figure 6). For this reason, the excitations observed here may not correspond to an IVCT or an LMCT, but to an internal ligand excitation. The Ti cations may provide only a charged “frame” for the π electronic system of the bridging ligand.

This interpretation has precedence in literature. Moilanen et al.^[9]^ reported about [(HAN){Mg(N,N’‐(2,6‐diisopropylphenyl)‐3,5‐dimethyldiketiminate)}_3_]⋅toluene complex reduced by [Mg(N,N’‐(2,6‐diisopropylphenyl)‐3,5‐dimethyldiketiminate)_2_]. In this compound three electrons were transferred to the ligand and caused absorptions at 800 nm and 920 nm. Calculation on the TD−B3LYP level confirmed that the observed spectra resulted from transitions from the highest occupied molecular orbital (HOMO) and a single occupied molecular orbital (SOMO) to the lowest unoccupied molecular orbital (LUMO). In this model compound the assignment was clear because no transition metals and d‐orbitals were involved in the coordination. However, up to six electrons of three TiCp_2_ complexes are available for a possible charge transfer in [(Cp_2_Ti)_3_HATN(Ph)_6_]. This significantly increases the complexity of the system comared to the system studied by Moilanen et al.^[9]^

In order to investigate the ground state of [(Cp_2_Ti)_3_HATN(Ph)_6_] more precisely, four spin multiplicities (*S*=0, 1, 2, 3) were calculated for the neutral species with PBE0. All multiplicities except *S*=3 show a strong spin contamination (SI‐3, Table S3). The spin states *S*={0, 1, 2} are also energetically degenerate. The mixing of higher spin states leads to assumption of a strong multireference character produced by the titanium centers, which is reasonable for such a highly symmetrical system. As reported in the literature, high spin contamination of the ground state wavefunction can lead to even higher spin contamination in the excited states and therefore lead to prediction of unphysical excitations.^[30]^ A single reference method should therefore not be able to describe such a complex accurately.

However, the experimental spectrum of [(Cp_2_Ti)_3_HATN(Ph)_6_] can still be calculated with sufficient accuracy using TD‐DFT in the spin states *S*=1 and *S*=2. Also, the spin states *S*=0 and *S*=3 are energetically very close to each other but show no electronic excitations in the NIR range. Table 3 shows that the experimental absorption signals are in exellent agreement with the theoretical simulations from an energetical perspective. Detailed discussion of the spectra will be provided in Section 2.4.

**Table 3:** NIR electronic excitations of measured spectrum and TD‐PBE0 excitations of [(Cp_2_Ti)_3_HATN(Ph)_6_].

Signal	Absorption energy [eV]		
	Experiment	TD‐PBE0 *S*=1	TD‐PBE0 *S*=2
0/I	0.60	0.56	0.55
0/II	0.95	0.92	0.93
0/IV	1.13	1.04	1.05
0/V	1.27	1.27	1.26

A correct description of electronic excitations with a spin‐contaminated wave function has already been observed in the literature.^[31]^ Nevertheless, the results of DFT shown here should be interpreted with reservations due to the high spin contamination of the system.

Looking at the structure of the orbitals involved in the excitation process (SI‐3, Figure S5), it becomes clear that only ligand‐ligand excitations are observed. As hypothesized above, a charge transfer from the titanium atoms to the ligand takes place in the ground state of [(Cp_2_Ti)_3_HATN(Ph)_6_], i. e. before any electronic excitation. Consequently, the titanium atoms are not involved in the electronic excitation process, but provide a charged frame for the excitation processes on the HATN(PH)_6_ ligand. Despite the good agreement of the theoretical excitations with experiment, the results are affected by spin degeneracy and spin contamination and therefore should be interpreted with caution. Both attributes indicate an intrinsic multi‐reference character. This character is caused by the titanium atoms and is not required to reproduce the observed NIR spectrum (cf. Section 2.5).

### 2.4 Calculation of Ground States and Electronic Transition Spectra of Other Redox States

**Figure 7:**
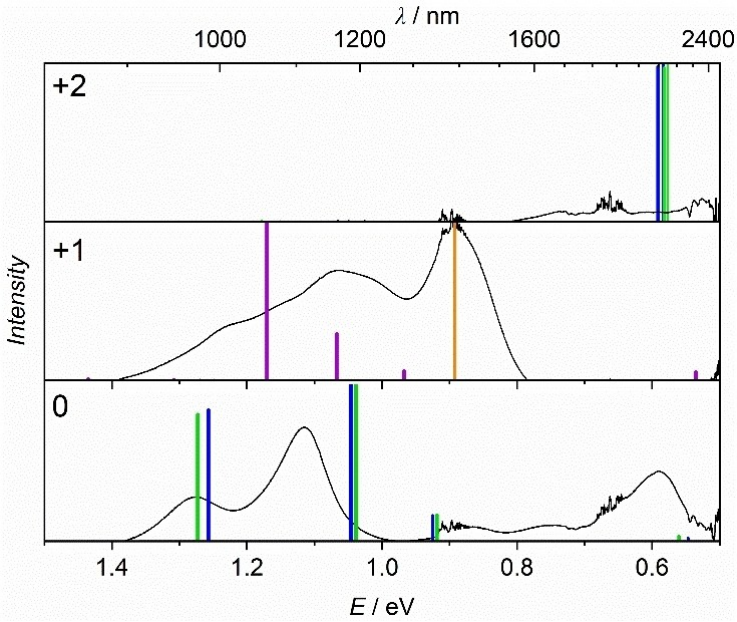
Comparison of experimental and theoretical NIR spectra for different charge states (0, +1, +2). Color scheme for multiplicities are orange: *S*=1/2, blue: *S*=1, green *S*=2, violet: *S*=3/2. Experimental data are referenced on the highest peak in the charge state +1. Theoretical species are normalized separately per redox species for a better comparison with experimental data. Transition dipole moments can be found in supporting information (SI‐3, Tables S3–S5).

Figure 7 shows, besides the NIR excitation of the charge state 0, the oxidized forms of the complex (+1, +2) in different spin states and the comparison to the experimental results. For all species at least three different multiplicities and their TD‐PBE0 spectra were calculated using the unrestricted Kohn‐Sham ansatz (see SI‐3, Table S3). The most important states were compared with experiment. Due to the high complexity of the system, no anionic species were computed in this work.

As mentioned before, the excitations of the neutral species from the experiment are well reflected by the TD‐DFT calculations. In both spin states (*S*=1 and *S*=2) it is possible to simulate the excitations observed in experiment. Only the broad signal around 2100 nm in Figure 7 cannot be reproduced in terms of intensity. Many factors can be considered for this behavior. For instance, the transition dipole moment depends on the selected functional and could be poorly described by PBE0. In addition, the spin contamination is highly significant as mentioned in Section 2.3., and the single‐reference approach cannot completely describe the real system.

However, a purely energetic consideration of the TD‐PBE0 spectra in comparison with experimental spectra leads to an excellent agreement. Besides that, a comparison of the different spin states shows quasi‐degenerate energies (SI‐3, Table S2). This observation suggests a multi‐reference character of the system, which will be discussed in more detail in Section 2.5. Also, the states *S*=0 and *S*=3 are energetically very close to each other but show no electronic excitations in the NIR range.

An examination of the redox species +1 shows that it is only possible to simulate the measured NIR spectrum if both, the S=1/2 (orange) and the S=3/2 (violet) spin states, are calculated and both spectra are overlaid. Similar to the neutral redox species, the total energies of both spin states are quasi‐degenerate and spin contamination is observed. This behavior also suggests a multi‐reference character. However, the transition energies of the theoretical excitation spectra correspond very well to the experimental data. This clearly shows that the measured results are only accessible by a combination of different spin states within the TD‐DFT framework. Only partial aspects of the electronic properties can be described with the single reference approach, which manifests itself in relevance of different spin states for the explanation of the observed UV/Vis‐spectra. A divergence in intensities, as observed in the neutral species, is also apparent. The charge species +2 shows no significant signals in experiment in Figure 4. No electronic excitation in the NIR range can be found in the theoretical calculations with the S=0 state. Only the spins S=1 and S=2 lead to excitations at ∼2000 nm in the theoretical studies. Similar to the neutral species, the first three spin states are energetically degenerate (SI‐3, Table S2). Again, the system is subject to a strong multi‐reference character. The signals seen in the calculations at about ∼2000 nm of the higher multiplicities cannot be recorded experimentally for two reasons: Firstly, the electronic excitations could be outside the accessible spectral range of the spectrometer if a deviation of 200 nm is assumed between calculated and experimental values. Secondly, another aspect is the poor description of a symmetric open shell system by a single‐reference method.

Nevertheless, the DFT can qualitatively present the electronic excitations in the NIR range, in which the Ti orbitals are not involved. However, this approach is not sufficient for a more detailed investigation of the system, since the spin degeneracy, which is triggered by a multi‐reference character, cannot be described well. The observations within TD‐DFT suggest that the multi‐reference character and the spin problem are probably located at the titanium atoms, since they are not involved in the excitations. Additionally, further complications can arise from the charged HATN ligand because of degenerate orbitals (Figure 5a). Therefore, the system will be treated within a multi reference framework below.

### 2.5 Interpretation of Magnetic Properties and Electronic Excitations Using Multireference Methods

**Figure 8:**
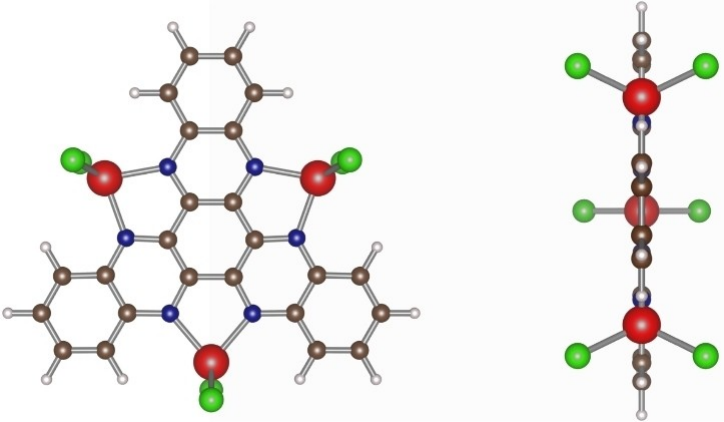
Simplified [(TiCl_2_)_3_HATN] complex. Color scheme of atoms is titanium: red, chlorine: green, carbon: brown, nitrogen: blue, hydrogen: white.

A key conclusion from Sections 2.3. and 2.4. is that the electronic transitions correspond to electronic excitations of the ligand and neither IVCT nor LMCT play a role in the electronic excitation. This also requires the ligand to accept electrons from the formally divalent Ti^II^ centers in the ground state. In order to explain how many electrons are transferred and which magnetic properties will result from such transfer, multi‐reference methods beyond DFT have to be used. In order to reduce the computational effort, a simplified model system (Figure 8) with similar electronic features is used for a CASSCF/NEVPT2 simulation with a CAS(6,7) space and a larger Def2‐TZVP basis set. Here, the Cp ligands are replaced by chlorine atoms and the phenyl groups of HATN(PH)_6_ are exchanged by hydrogen atoms. This approach was already used successfully in the literature for other systems.^[32]^ In order to validate the justification of the simpler model system a direct comparison of the TD‐DFT spectra with the original complex is shown in SI‐3, Figure S6.

Magnetic measurements have shown that up to three unpaired electrons with parallel spin can be present in the system.^[12]^ The calculations in Section 2.3. demonstrate that the charged HATN ligand without coordinated metals can reproduce the NIR electronic excitation provided that up to three electrons are transferred from the metal framework to the ligand in the ground state. For such a transfer to the HATN ligand, six electrons of the three formally titanium(II) centers are available.

**Figure 9:**
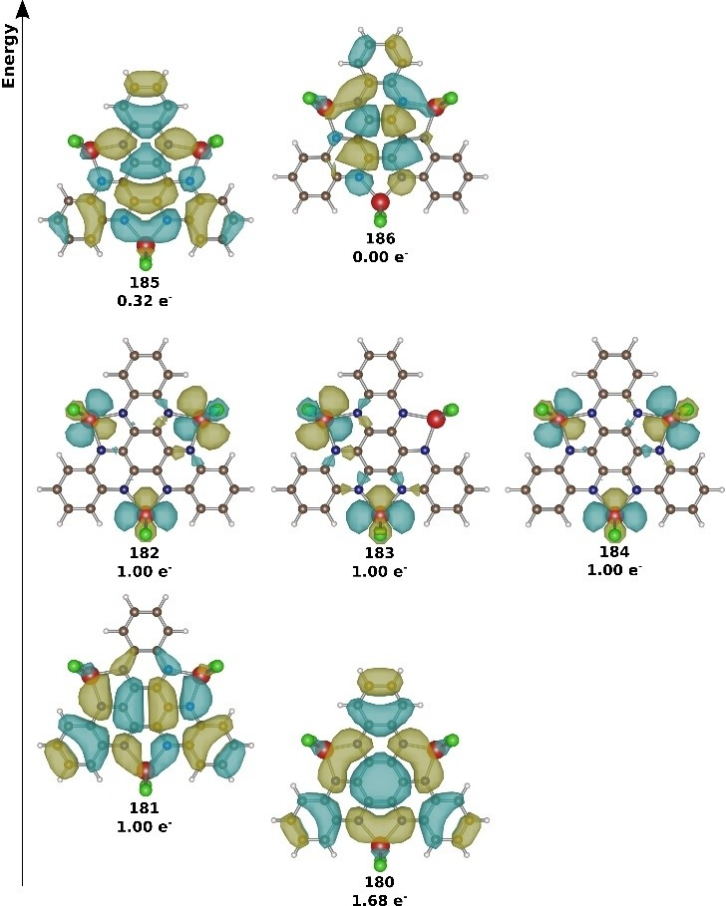
Visualization of molecular orbitals in a canonical representation and its occupation within CAS(6,7) for *Q*=0, *S*=1 as representative state. Color scheme of atoms is titanium: red, chlorine: green, carbon: brown, nitrogen: blue, hydrogen: white. Orbitals are labeled similar to calculated output.

A proper description of the multi‐reference character and the electronic excitation in the NIR range requires at least a (6,7) space for this system. Starting from *Q*=0, *S*=3 pbe0 wavefunction, it can be observed that three singly occupied titanium d‐orbitals and three singly occupied π orbitals arise. Therefore, a CAS(6,6) calculation provides a good ansatz for the ground state. According to the D_3h_ point group symmetry of the system, two energetically degenerate states are formed, which consist of a variety of configuration state functions. In order to include possible electronic excitations, another virtual orbital is taken into the active space. A further unoccupied ligand orbital is found to be necessary for the electronic excitation resulting in a CAS(6,7) active space. If the individual states are reduced to the dominant determinants and Löwdin Reduced Orbitals are formed, a formal description can be made in a one‐electron picture with fractional occupation numbers (Figure 9).

A total of five orbitals are occupied in the electronic ground state, where three singly occupied orbitals (182, 183, 184) are strongly localized at the titanium atoms. Additionally, one singly occupied (181) and a doubly occupied (180) orbital is found at the HATN ligand. The doubly occupied HATN ligand orbital (180) exhibits a population of 1.68 e^−^ and delivers electron density to a higher lying HATN ligand orbital (185). A comparison of the populated HATN ligand orbitals with the orbitals of a free HATN molecule shows that the LUMO and LUMO+1 orbitals were occupied after the charge‐transfer from the metal centers (SI‐3, Figure S4 and Figure 9). The titanium atoms share three electrons in this picture which are distributed over all three centers. These electrons are responsible for the strong multi‐reference character and lead to a spin degeneracy reminiscent to the single reference calculations (SI‐3, Table S6–S7). These results can also give first insights into the magnetic measurements of the unpaired electrons.^[12]^ Furthermore, it can be concluded that three electrons were donated to the HATN ligand in the ground state (before electronic excitation) and that there are on average three equivalent Ti^III^ centers. This observation could be verified with CAS(12,13) and a fully geometry‐symmetrized model system which leads to similar results even when trying to rotate more Ti‐centered molecular orbitals in the active space (SI‐3, Table S8 and S15).

The electronic excitation in the framework of the multi‐reference picture displays a similar behavior as the single reference TD‐DFT spectra. With both methods, the NIR region is solely dominated by internal HATN ligand orbitals, and the electrons localized at the titanium atoms are not involved in any excitation. However, a specific transition cannot always be interpreted with a one‐electron picture. For this reason, an attempt is made to use the dominant configuration state functions to obtain an approximate one‐electron image. Table 4 shows the respective excitation energies at CAS(6,7)/NEVPT2 level compared to the TD‐PBE0 energies. In addition, the dominant configurations of the individual states at each transition are shown. Note, that the shown energies for CAS(6,7)/NEVPT2 are provided by the optimized ground state wavefunction and no state averaging or state optimizing of the excited states was performed due to the high complexity of configurations (SI‐5, Table S16).

**Table 4:** Electronic excitations with TD‐PBE0, CAS(6.7)/NEVPT2 (*Q*=0, *S*=1) and the deviation of both methods. The right columns represent the dominant transitions of the excited states of CAS(6,7) calculations. A full configuration of the excited states is provided in supporting information (SI‐5, Tables S16 and S17).

Excitation energies [eV]			Dominant transitions		
TD‐PBE0	CAS(6,7)/ NEVPT2	Deviation [eV]	orbital x	→	orbital Y
0.57	0.43	0.14	180	→	181
0.89	0.74	0.15	180	→	185
1.15	1.28	−0.13	180	2x→	185
1.50	1.65	−0.15	181	→	186

Obviously, the excitation energies agree reasonably well between both methods. Three of the most important excitations can be represented by a single transition. The energetically lowest (0.44 eV) can be interpreted as an excitation from the almost doubly occupied orbital 180 (1.68 e^−^) to the singly occupied orbital 181 (1.00 e^−^). At 0.77 eV the dominant configuration is a transition from orbital 180 to orbital 185 (0.32 e^−^), which is already slightly occupied. The energetic largest transition at 1.65 eV can be interpreted as an excitation from orbital 181 (1.00 e^−^) into the unoccupied orbital 186. The signal at 1.28 eV can no longer be interpreted within a simple one‐electron excitation in the orbital picture, since the dominant configuration state functions (only ∼10 %) represent a double transition from orbital 180 (1.68 e^−^) to orbital 185 (0.32 e^−^). The state shows a large multi‐reference character with up to seven dominant determinants with over 5 % weight. The corresponding configuration is listed in SI‐5, Table S16. Please note, that a double excitation cannot be calculated with TD‐DFT and a direct comparison of this excitation can only be made with reservation.

## 3 Conclusions

Three reduction waves and six oxidation waves are observed in voltammograms of [(Cp_2_Ti)_3_HATN(Ph)_6_]. All electron transfers are one electron processes clearly separated on the potential scale. The solution spectra of [(Cp_2_Ti)_3_HATN(Ph)_6_] and its oxidation and reduction products [(Cp_2_Ti)_3_HATN(Ph)_6_]^3−^, [(Cp_2_Ti)_3_HATN(Ph)_6_]^2−^, [(Cp_2_Ti)_3_HATN(Ph)_6_]^1−^, [(Cp_2_Ti)_3_HATN(Ph)_6_]^1+^ and [(Cp_2_Ti)_3_HATN(Ph)_6_]^2+^ show electronic transitions in the UV, vis and NIR spectral ranges. DFT calculations on TD‐DFT and CASSCF/NEVPT2 levels show that three electrons are transferred from the formally titanium(II) centers to the bridging ligand in the ground state. However, the complete spectra can only be explained when assuming that spin states of different multiplicity form the ground state of [(Cp_2_Ti)_3_HATN(Ph)_6_]. The observed electronic excitations in the UV, Vis and NIR ranges are all transitions between ligand levels. Contrary to common notion, LMCT or IVCT transitions do not play a role. The same assignment rules can also be reproduced by calculating the isolated and appropriately charged HATN ligand. The temperature‐dependent magnetic properties can be explained the hybridization of different electronic configurations of different total spin with one valence electron located at each of the three Ti centers and one electron in a SOMO of the ligand.

## Experimental Section

### Substances

[(Cp_2_Ti)_3_HATN(Ph)_6_] was synthesized as reported.^[12]^ All used solutions were freshly prepared from thoroughly dried solvents. The electrolytes were made with tetrabutylammonium hexafluorophosphate (TBAPF_6_), Sigma Aldrich, Steinheim, Germany), silver perchlorate (AgClO_4_, Sigma Aldrich). Acetonitrile (MeCN) and tetrahydrofurane (THF) were dried according to standard procedures^[33]^ prior to use and stored under nitrogen. [(Cp_2_Ti)_3_HATN(Ph_)6_] was solved in dry THF to a concentration of 0.3 mM.

### Instrumentation

Electrochemical and spectroscopic setups have been installed in a glovebox and connected to instruments outside the box via gas‐tight integration of cables and optical fibers into a flange connector (M. Braun). Further details are given in SI‐1. NIR spectra were measured with a fiber‐coupled Matrix‐F FT‐NIR spectrometer (Bruker Optik GmbH, Ettlingen, Germany). UV‐Vis spectra were taken with a GetSpec 2048CCD array spectrometer (GetSpec, Sofia, Bulgaria). Both spectrometers were coupled to a spectroelectrochemical cell (ALS Co., LTD, Tokyo, Japan) located inside an Ar‐filled glove box using 600 μm diameter N227 quartz fibers (Bruker Optik GmbH).

Cyclic voltammograms (CV) and differential pulse voltammograms (DPV) were recorded at 295 K using a potentiostat (Compactstat, Ivium Technologies, Eindhoven, The Netherlands) with a three‐electrode assembly consisting of an Au disc as working electrode (WE, diameter *d*=2 mm), a Pt plate as auxiliary electrode (Aux, *A*=1 cm^2^) and a Ag/Ag^+^ reference electrode filled with 0.01 M AgClO_4_ and 0.1 M TBClO_4_ in THF.

The formal potentials *E°*’ were obtained as the arithmetic mean of the anodic *E*_pa_ and cathodic peak potentials *E*_pc_. They are quoted with respect to the ferrocene/ferrocenium redox couple (Fc/Fc^+^) which was measured in solution with same concentration of supporting electrolyte and the same concentration of the Fc (0.3 mM) as the compound under investigation.

### Quantum chemical calculations

All calculations were performed using the program package ORCA in version 4.2.^[34]^ The basis set Def2‐SVP is taken in all simulations with the [(Cp_2_Ti)_3_HATN(Ph)_6_] complex and the pure HATN ligand.^[35]^ The simplified model system is calculated using a Def2‐TZVP basis set. For the calculation with the single‐reference properties, the hybrid density functional PBE0 an unrestricted open‐shell ansatz was selected. Due to the large number of electrons, the Resolution‐of‐Identity formalism RIJCOSX was used with a Def2/J auxiliary basis set as implemented in ORCA.^[36]^ For the respective redox species, four different spin multiplicities were geometry‐optimized and subsequently TD‐DFT calculations for the UV/Vis spectra were performed. For a verification of these spectra, a CAS(6,7)/NEVPT2 and a CAS(12,13) calculation for the smaller model system was carried out. Due to the large basis set, RIJCOSX with a Def2/JK auxiliary basis set was selected. The calculated wavefunction for CAS(6,7) was state‐averaged over the first two states to generate the correct degenerate ground state. No state optimizations for the excited states were performed due to the high complexity of the system.

## Conflict of interest

The authors declare no conflict of interest.

## Supporting information

As a service to our authors and readers, this journal provides supporting information supplied by the authors. Such materials are peer reviewed and may be re‐organized for online delivery, but are not copy‐edited or typeset. Technical support issues arising from supporting information (other than missing files) should be addressed to the authors.

Supplementary
