# Supplementary material for: Electronic Transitions in Different Redox States of Trinuclear 5,6,11,12,17,18‐Hexaazatrinaphthylene‐Bridged Titanium Complexes: Spectroelectrochemistry and Quantum Chemistry
Source: Chemphyschem. 2020 Oct 16;21(22):2506–14. doi: 10.1002/cphc.202000547 (PMC7756296; doi:10.1002/cphc.202000547)
Supplement: Supplementary file 1 — Supplementary [file CPHC-21-2506-s001.pdf]

ChemPhysChem

Supporting Information

Electronic Transitions in Different Redox States of Trinuclear 5,6,11,12,17,18-Hexaazatrinaphthylene-Bridged Titanium Complexes: Spectroelectrochemistry and Quantum Chemistry

Aleksandra Markovic+, Luca Gerhards+, Pia Sander, Carsten Dosche, Thorsten Klüner, Rüdiger Beckhaus, and Gunther Wittstock*

SI-1 Instrumentation

NIR spectra were measured with a fiber-coupled Matrix-F FT-NIR spectrometer (Bruker Optik GmbH, Ettlingen, Germany) equipped with a halogen lamp as NIR source and an InGaAs detector. Spectra were taken in the range from 12000 to 4000 cm^{-1} with a resolution of 8 cm^{-1}. For each measurement 16 scans were accumulated. UV-Vis spectra were taken with a GetSpec 2048 CCD array spectrometer (GetSpec, Sofia, Bulgaria). The device configuration allowed a resolution of 2.4 nm in the range of 200 to 1000 nm. For the measurements, an integration time of 100 ms was used. Both spectrometers were coupled to a spectroelectrochemical cell (ALS Co., LTD, Tokyo, Japan) located inside an Ar-filled glove box (Figure S1) using 600 µm-diameter N227 quartz fibers (Bruker Optik GmbH).

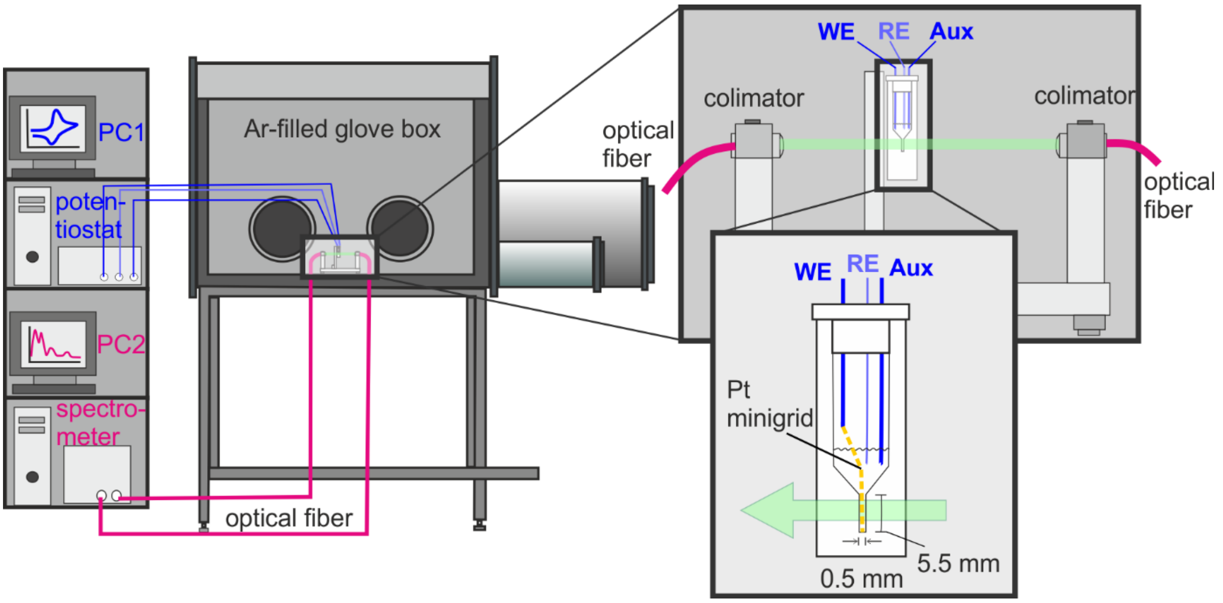

Figure S1. Experimental setup for spectroelectrochemical measurements.

SI-2 Attempted interpretation of the electronic spectra within single electron model

Attempted orbital model and assignment of transitions

Despite very coarse assumptions required and their known severe limitations,[1] we attempted an assignment of all transitions in an experimentally derived single electron (orbital) picture for all redox states using the formal potential *E*°' of the electron transfer reactions from Table 1 and the energies of the electronic transitions in Figure 4. This attempt is documented here. Despite its the perceived inner consistency, this assignment could not be reproduced by quantum chemical calculations due to the inherent limitations of the MO model discussed in 2.2 and the consequences of the coarse assumptions required.

The correlation between the spectroscopic and electrochemical data in a semi-quantitative approach involved

i) Definition of a ground state configuration using experimental solid-state magnetic data
ii) The definition of a Fermi energy for dispersed molecular systems
iii) Interpretation of observed electronic transitions within a single electron picture, i.e. equating the spectroscopically observed energy of the electronic transition as the difference between two orbital energies
iv) Definition of a "Fermi energy" of molecular systems as the mean between HOMO and LUMO or to the value of a SOMO. This energy is equated with the Fermi energy from *ii*)
v) Recalculation of the electrochemical data to the vacuum level

Definition of the ground state configuration (*i*). The general MO scheme is derived from common textbook MO schemes, where metal orbitals and ligand orbitals form MOs, in which the metal-centered d-type orbitals are the frontier orbitals (2 e_g and 3 t_g orbitals for tetrahedral complexes).[2] The highest occupied ligand π orbital (referred to as HOMO in the following schemes) is the first orbital below the d-e_g set and the lowest unoccupied π^* orbital of the ligand (referred to as LUMO) represents the first orbital above the d t_2 set. Note that the MO-scheme of the HATN-ligand as represented schematically in Figure 5 has already been simplified in this approach.
For the neutral complex it is known from magnetic susceptibility measurements that this compound is not a closed shell system in respect to the ligand.[3] At least one electron has to be transferred from one Ti(II) center to a ligand orbital, forming a Ti(III) center and one ligand-centered SOMO. Based on the irreversibility of the reduction of $HATN^-$, the transfer of only one electron has been assumed, although a transfer of more than one electron would be an option. Ground states of other redox states were derived from this ground state by subsequent addition or removal of electrons. Note that theoretical calculations in section 2.3-2.5 of the main manuscript showed that actually all metal centers of the neutral complex are Ti(III).

Definition of Fermi levels for dispersed molecules (*ii*). In case two redox forms of one molecular system are dissolved in an electrolyte, the "Fermi level of electrons" can be set to the Nernst-Potential of an inert electrode in this solution[4] and recalculated to the vacuum level (*vi*). In our case, one redox form of a complex with total charge *z*+ is dissolved in an electrolyte solution. This metal complex can undergo a one-electron oxidation with a formal potential $E°'(X^{+z}/X^{+(z+1)})$. The complex can also undergo a one-electron reduction with a formal potential $E°'(X^{+(z-1)}/X^{+z})$. The Fermi energy of the redox state X^{+n} on the electrochemical

potential scale is than taken as midterm potential $E_m = ½[E°′(X^{+(z-1)}/X^{+z}) + E°′(X^{+z}/X^{+(z+1)})]$ between two adjacent one-electron oxidation and one-electron reduction in the cyclic voltammogram. At the potential E_m, there is an equal probability that the complex accepts an electron from an inert electrode or transfers an electron to the electrode. The concentrations of the adjacent redox states are exactly equal, provided the oxidation and reduction is kinetically fast, which is the case in our system.

Equating the energy of the spectroscopy transition to the difference of orbital energies (*iii*). This is a consequence of assuming a single electron picture for the interpretation of the spectroscopic transitions. In that picture transitions can be interpreted as electron transitions between discrete orbitals.

The starting point for assignment of transitions between ligand orbitals is the energy spacing between HOMO, LUMO and if applicable SOMO. The energetic position of the SOMO within the MO scheme is fixed by the π-π^*, π-π^r and π^r-π^* transitions. Typically, those electronic transition can be identified in the experimental spectra due to their characteristic shape. The index r marks transitions involving single occupied (**r**adical) orbitals because these show special spin selection rules. Please, note that the SOMO may be filled or emptied in other redox states. The transitions which could not be assigned to this set of π orbitals were assumed to be charge transfer transitions between ligand and metal centers. The energies of the d-type orbitals are then derived from these assumed charge transfer bands. However, this empirical approach does not guarantee for correctness of the assumption as the quantum chemical calculations illustrate for $[(Cp_2Ti)_3HATN(Ph)_6]$.

Definition of a "Fermi energy" of molecular systems as the mean between HOMO and LUMO (*iv*). The "Fermi energy" is taken as the mid-value between HOMO and LUMO, or as the energy of the SOMO. This value is equated to the value derived in *ii*) from electrochemical data and is used to place energy levels of different redox states on an absolute scale.

Recalculation of the electrochemical data to the absolute energy scale (*v*). The assessment of the spectroscopic data provides the relative orbital energies for one oxidation state, whereas the electrochemical data show the relative shift of the orbital sets if one compound is reduced or oxidized. To provide a complete picture for comparison of all redox states, the relative energies are referred to the vacuum level. The electrode potentials are measured against the ferrocene/ferrocenium (Fc/Fc^+) redox couple, which is referenced to the standard hydrogen electrode by

$$E°′(Fc/Fc^+) = (0.400 \pm 0.005)\ \text{V vs. SHE.}^{[5]} \quad (S1)$$

The electrode potentials against the SHE are than recalculated against the SHE scale according to the IUPAC recommendation[6]

$$E_{vac} = -e \cdot E_{SHE} - (4.44 \pm 0.02)\ \text{eV}. \quad (S2)$$

Combining Eq (S1) and (S2) yields

$$E_{vac} = -e \cdot E_{Fc/Fc+} - (4.84 \pm 0.025)\ \text{eV}. \quad (S3)$$

Assignment of transitions for each charge state

Below the qualitative assignment is detailed, which is summarized in Figure S2. The codes for the transitions are from Figure 4.

Charge state 0. The assumed electronic structure of the neutral complex allows several characteristic electronic transitions. The UV-vis part of the spectra is dominated by the π-π* transition from the HOMO to the LUMO orbital of the ligand at around 3.0 eV (0/X). The energy of this transition does not change significantly between the different redox states. In the UV-vis region, there are also the π-π^r transition from the HOMO to the SOMO orbital at around 1.9 eV (0/VI), ligand to metal charge transfer (LMCT) from the HOMO orbital of the ligand to Ti(t) at around 2.3 eV (0/IX), and the degenerate metal to ligand charge transfer (MLCT) from the Ti(II)(e) to the LUMO orbital of the ligand at around 2 eV (0/VII, 0/VIII). For the same oxidation state, the NIR region of the spectra shows the π_r-π* transition from the SOMO to the LUMO orbital at 1.1 eV (0/IV), LMCT at 0.6 eV (0/I) and 0.75 eV (0/II) and the MLCT from the Ti(II)(e) to the SOMO orbital of the ligand at around 0.9 eV (0/III). The signals at 0.68 eV (*) and 0.9 eV (*) are experimental artifacts caused by overtones of molecular vibration of the solvent, which are not fully compensated during measurement. They appear in each of the NIR spectra in Figure 4. Transition 0/V is a vibronic progression of 0/IV corresponding to C=C valence vibration (~1600 cm^{-1}).

Charge state +1. After a one-electron oxidation, an electron is removed from the SOMO orbital which becomes the new LUMO orbital (denoted as LUMO' in Figure S2). This charge state has two π-π* transitions from the HOMO to the LUMO' and the LUMO orbital at around 2.5 eV (+1/V) and 2.9 eV (+1/VI). The NIR part of the spectra is characterized by two MLCT from the Ti(II)(t) and Ti(III)(t) to the LUMO' at 0.9 eV (+1/I) and 1.1 eV (+1/II), and two MLCT from the Ti(II)(t) and Ti(III)(t) to the LUMO at 1.05 eV (+1/III) and 1.25 eV (+1/IV).

Charge state +2. In this charge state, an electron is removed from the one Ti(II)(t). All other transitions are observed as in the charge states discussed before. These transitions and the participating states are slightly shifted to lower energies. The MLCT cannot be seen clearly in the NIR part of the spectra, there is only a general rise in absorption in the spectra which indicate those transitions.

Charge state -1. After the first one electron reduction (overall charge 0 to -1), an electron is added to the Ti(III)(t) orbital, after which no mixed valence systems exists. In the NIR part of the spectra, one can see the MLCT at 0.75 eV (-1/I), the π^r-π* at 1.1 eV (-1/II). The LMCT from the SOMO orbital of ligand to the Ti(t)(II) orbital is not observed in the recorded wavelength range, but one can see the rise in absorption at low photon energies which indicates that there is the transition in the energy range below 0.5 eV. In the UV-vis range, there are 3 transitions: the π-π* at 2.8 eV (-1/VI), the π-π^r at 1.7 eV (-1/IV) and the LMCT from the HOMO orbital of the ligand to the Ti(t)(II) orbital at 2.3 eV (-1/V).

Charge state -2. After the second one electron reduction (overall charge state -1 to -2), an electron is transferred into the SOMO orbital of the ligand and this orbital becomes the new HOMO orbital (denoted as HOMO' in Figure S2). In this charge state, some transitions are lost. One can still see two π-π* transitions from the HOMO and the HOMO' to the LUMO orbital at 2.6 eV (-2/V) and 0.8 eV (-2/I) as well as the MLCT from the Ti(e)(II) to the LUMO orbital at 1.7 eV (-2/III). The LMCT from the HOMO to the Ti(t)(II) is at 2.2 eV (-2/IV). However, the LMCT from the HOMO' to the Ti(t)(II) cannot be observed, yet there is a rise in absorption which indicate that there is the transition at energies below 0.5 eV. In the third reduction, an electron is accepted by one of the Ti centers. The complex is then composed of one Ti(I) and two Ti(II) centers. The transitions are similar to that of the charge state -2. The only difference is the MLCT from the Ti(e)(II) to the LUMO orbital.

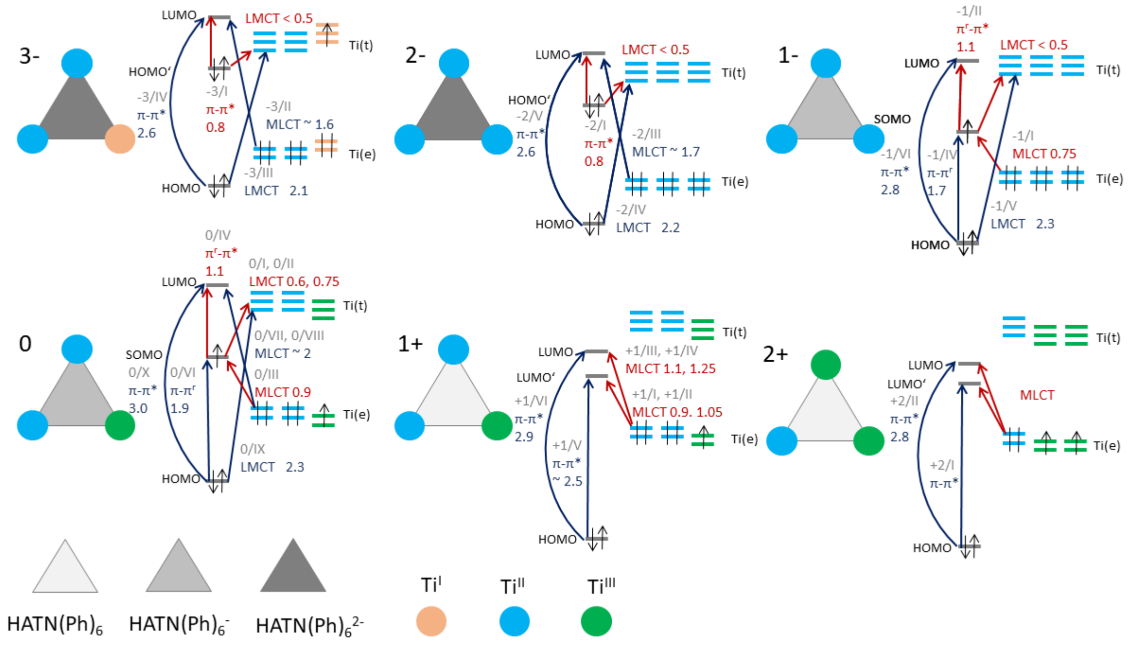

Figure S2. Orbital energy diagram and transitions (in eV) observed in electronic spectra in the visible and NIR range for $[(Cp_2Ti)_3HATN(Ph)_6]$ and its oxidation products ($[(Cp_2Ti)_3HATN(Ph)_6]^{1+}$, $[(Cp_2Ti)_3HATN(Ph)_6]^{2+}$) and reduction products ($[(Cp_2Ti)_3HATN(Ph)_6]^{3-}$, $[(Cp_2Ti)_3HATN(Ph)_6]^{2-}$, $[(Cp_2Ti)_3HATN(Ph)_6]^{1-}$). Transitions involving single occupied ligand orbital are marked with π^r (**r**adical). As spins are parallel within one Ti center but may be antiparallel between different Ti centers, d electron spins are indicated as II.

Construction of an Energy Diagram

From the assignments in Figure S2 and the spectra (Figure 4 of the main manuscript) one can construct the “orbital diagram” in an energy scale[7] (Figure S3). For this purpose, the data from the electrochemical experiments are transformed to the absolute energy scale by Eq. (S3). From the assignments Figure S3, it is evident that the positions of Ti-centered orbitals are not affected if an electron is removed from the SOMO (HOMO’, LUMO’) orbital, which seems to be purely ligand-centered. However, if electrons are instead removed from the Ti centers the energetic position of all orbitals in the complex is affected indicating an interaction of the d-orbitals with the ligand.

When the complex is oxidized from -3 to -2 charge state, an electron is removed from antibonding t orbital (because of simplicity all t_2 orbitals are denoted in the text as t orbitals), and this influences all orbitals by shift of -0.5 eV. In the next oxidation (from -2 to -1), the electron is removed from the π-system (HOMO’ becomes SOMO). The Ti-centered orbitals do not change energy because these orbitals are not interacting with the HOMO’ (now SOMO). In next oxidation, an electron is removed from the Ti-centered orbital and, because of π-back bonding, all orbitals are shifted by about -0.5 eV. In the ground state the complex is in the triplet state because of electron paring of two single occupied orbitals (Ti(III)(e) and SOMO). When the titanium complex is oxidized from 0 to +1, removing an electron from the SOMO orbital (now LUMO’) and again the titanium centered orbitals do not change energy. The energy difference between LUMO and LUMO’ becomes smaller because one unpaired electron is removed from the SOMO orbital, and the complex is again in the doublet state. In oxidation from +1 to +2 charge state, an electron is removed from a Ti-centered orbital and because of π-back bonding, all orbitals are shifted by -0.5 eV to lower energies.

Calculation of orbital diagrams. The midterm potential (E_m) is taken as the arithmetic mean of two neighboring redox potentials determined from DPV measurement (vs. Fc/Fc^+), (Figure 2 of main manuscript). It indicates a potential at which a certain charge state of the complex is stable, i.e. an oxidation to the next higher or a reduction to a lower charge state are equally likely. In the absence of a SOMO orbital in a certain charge state of the complex, this value is taken to be equal to the mean value of the LUMO and the HOMO in that charge state. In the presence of a SOMO orbital, this value corresponds to the energy of SOMO orbital. After this assignment which places the different charge states relatively to each other, the remaining orbital positions were constructed using transition energies from absorption spectra. Orbitals which do not contribute to an electronic transition were omitted from orbital diagrams.

The orbitals of Ti(III) centers have lower energies than those of Ti(II) because removing further electrons from the same metal center becomes increasingly difficult when electrons have already been removed before. The energetic difference between the Ti(III) and the Ti(II) orbitals is always the same and calculated to be 0.16 eV from the optical transition of the neutral complex.
The energetic difference between the Ti(t) and the Ti(e) was taken to be 1.65 eV. This value was calculated from data of the neutral complex.

Charge state 0.
The E_m was determined to be -1.34 V vs. Fc/Fc^+ or 3.50 eV vs. Vac according to Eq. (S3). This is the energy of the SOMO.

Calculation from absorption spectra
LUMO (-2.40 eV) = SOMO (-3.50 eV) + π^r-π^* (1.10 eV, 0/IV)
HOMO (-5.40 eV) = SOMO (-3.50 eV) - π-π^r (1.90 eV, 0/VI)
Ti(II)(e) (-4.40 eV) = SOMO (-3.50 eV) - MLCT (0.90 eV, 0/III)
Ti(II)(t) (-2.75 eV) = SOMO (-3.50 eV) + LMCT (0.75 eV, 0/II)
Ti(III)(t) (-2.90 eV) = SOMO (-3.50 eV) + LMCT (0.60 eV, 0/I)
Ti(III)(e) (-4.56 eV) = Ti(II)(e) (-4.40 eV) - 0.16 eV

Charge state +1.
The E_m was determined to be -0.785 V vs. Fc/Fc^+ or -4.05 vs Vac according to Eq. (S3). This value of -4.05 eV corresponds to the mean value between the lowest unoccupied (in this case LUMO’) and the highest occupied level (Ti(II)(e)).

Calculation from absorption spectra
The transition between LUMO’ and the Ti(II)(e) is the MLCT (0.90 eV, +1/I). From this information the energy of the LUMO’ (-3.60 eV) and the Ti(II)(e) (-4.50 eV) was calculated
Ti(II)(e) (-4.50 eV) = Ti(III)(e) (-4.66 eV) + 0.16 eV
Ti(III)(t) (-3.00 eV) = Ti(III)(e) (-4.66 eV) + 1.65 eV
Ti(II)(t) (-2.85 eV) = Ti(II)(e) (-4.50 eV) + 1.65 eV
HOMO (-6.10 eV) = LUMO’ (-3.60 eV) - π-π^* (2.50 eV, +1/V)
LUMO (-3.20 eV) = HOMO (-6.10 eV) + π-π^* (2.90 eV, +1/VI)

Charge state +2.

The E_m was determined to be -0.315 V vs. Fc/Fc$^+$ or -4.52 vs. Vac according to Eq. (S3). The value of -4.52 eV corresponds to the mean value between the lowest unoccupied (LUMO') and the highest occupied level (Ti(II)(e)).

Calculation from absorption spectra
The transition between LUMO and the Ti(II)(e) is the MLCT. Since the exact values of the MLCTs are not known for this state, approximation was made that the MLCT has the same value as in +1 charge state, this laces the Ti(II)(e) (-4.97 eV) and the LUMO' (-4.07 eV).

Ti(III)(e) (-5.12 eV) = Ti(II)(e) (-4.97 eV) - 0.16 eV
Ti(II)(t) (-3.32 eV) =Ti(II)(e) (-4.97 eV) + 1.65 eV
Ti(III)(t) (-3.47 eV) = Ti(III)(e) (-5.12 eV) + 1.65 eV
HOMO (-6.57 eV) = LUMO' (-4.07 eV) - π-π*(2.50 eV, +2/I)
LUMO (-3.77 eV) = HOMO (-6.57 eV) + π-π*(2.80 eV, +2/II)

Charge state -1.
The E_m was determined to be -1.840 V vs. Fc/Fc$^+$ or -3.00 eV vs. Vac according to Eq. (S3). This is the energy of the SOMO.

Calculation from absorption spectra
LUMO (-1.90 eV) = SOMO (-3.00 eV) + $π^r$-π*(1.1 eV, -1/II)
HOMO (-4.70 eV) = SOMO (-3.00 eV) - π-$π^r$ (1.7 eV, -1/IV)
Ti(II)(e) (-3.75 eV) = SOMO (-3.00 eV) - MLCT (0.75 eV, -1/I)
Ti(II)(t) (-2.10 eV) = Ti(II)(e) (-3.75 eV) + 1.65 eV

Charge state -2.
The E_m was determined to be -2.44 V vs. Fc/Fc$^+$ or -2.40 eV vs. Vac according to Eq. (S3). The value of -2.40 eV corresponds to the mean value between the lowest unoccupied (Ti(II)(t)) and the highest occupied level (HOMO').

Calculation from absorption spectra
The transition between Ti(II)(t) and HOMO' is the LMCT. Energy of this transition is lower than 0.5 eV (in this case it was taken the approximation that this transition is at 0.4 eV). From this information the energy of the HOMO' (-2.60 eV) and the Ti(II)(t) (-2.20 eV) was calculated.

Ti(II)(e) (-3.85 eV) = Ti(II)(t) (-2.20 eV) - 1.65 eV
HOMO (-4.40 eV) = Ti(II)(t) (2.20 eV) - LMCT (2.20 eV, -2/IV)
LUMO (-1.80 eV) = HOMO (-4.40 eV) + π-π* (2.60 eV, -2/V)
At the end the approximation was checked by:
LUMO (-1.80 eV)-π-π* (0.80 eV, -2/I) = HOMO' (2.60 eV)

Charge state -3.
For this charge state it was approximated that the E_m was -2.97 V vs. Fc/Fc$^+$ or -1.87 eV vs. Vac according to Eq. (S3). This approximation is based on the observation that the potential difference between the $E°´$ values of the 0/-1, -1/-2, -2/-3 redox pair is very similar. This differences were averaged and half of that difference was subtracted from E°'(-2/-3) to arrive at an estimation of E_m for the -3 charge state. The value of -1.87 eV corresponds to the middle value between the lowest unoccupied (Ti(II)(t)) and the highest occupied level (HOMO').

Calculation from absorption spectra.
The transition between these two levels is the LMCT which energy is lower than 0.5 eV (in this case it was taken the approximation that this transition is at 0.3 eV). From this information the energy of the HOMO’ (-2.02 eV) and the Ti(II)(t) (-1.72 eV) was calculated.

Ti (II)(e) (-3.37 eV) = Ti(II)(t) (-1.72 eV) - 1. 65 eV
HOMO (-3.82 eV) = Ti(II)(t) (-1.72 eV) - LMCT (2.1 eV, -3/III)
LUMO (-1.22 eV) = HOMO (-3.82 eV) + π-π*(2.6 eV, -3/IV)

At the end the approximation was checked by:
LUMO (-1.22 eV)-π-π*(0.8 eV, -3/I) = HOMO’(-2.02 eV)
This value matched the value of the HOMO’ calculated before.

Table S1. Energy levels [eV vs. vacuum potential scale] of the complex (purple values indicate approximated values, red numbers in the SOMO column are actually the mean value between the LUMO and the HOMO of a certain charge state.

	SOMO	LUMO	HOMO	Ti(I)(t)	Ti(II)(t)	Ti(III)(t)	Ti(I)(e)	Ti(II)(e)	Ti(III)(e)	HOMO'	LUMO'
0	-3.50	-2.40	-5.40	/	-2.75	-2.90	/	-4.40	-4.56	/	/
+1	-4.05	-3.20	-6.10	/	-2.85	-3.00	/	-4.50	-4.66	/	-3.60
+2	-4.52	-3.77	-6.57	/	-3.32	-3.47	/	-4.97	-5.12	/	-4.07
-1	-3.00	-1.90	-4.70	/	-2.10	/	/	-3.75	/	/	/
-2	-2.40	-1.80	-4.40	/	-2.20	/	/	-3.85	/	-2.60	/
-3	-1.87	-1.22	-3.82	-1.57	-1.72	/	-3.22	-3.37	/	-2.02	/

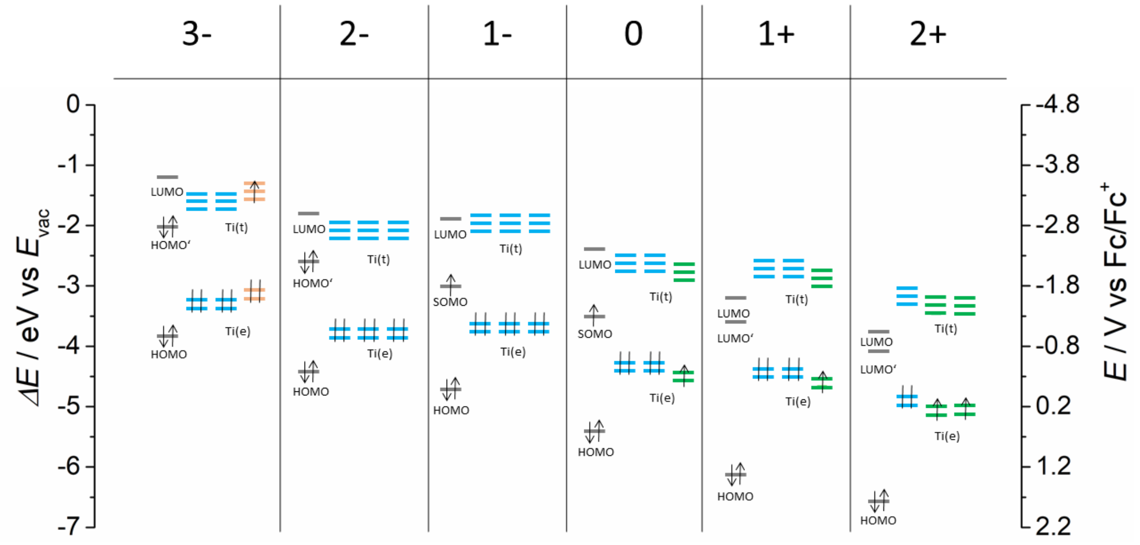

Figure S3. The energy accurate orbital diagram of the $[(Cp_2Ti)_3HATN(Ph)_6]$ and its oxidation products ($[[(Cp_2Ti)_3HATN(Ph)_6]^{1+}$, $[(Cp_2Ti)_3HATN(Ph)_6]^{2+}$) and reduction products ($[[(Cp_2Ti)_3HATN(Ph)_6]^{3-}$, $[(Cp_2Ti)_3HATN(Ph)_6]^{2-}$, $[(Cp_2Ti)_3HATN(Ph)_6]^{1-}$). The charge state of the complex is stated in the first row of the table. The energy is referenced to the vacuum level on the left ordinate and to the formal potential of the Fc/Fc^{+} redox couple on the right ordinate. As spins are parallel within one Ti center but may be antiparallel between different Ti centers, d electron spins are indicated as II.

The orbital picture discussed above is not a good approximation of the ground state of $[(Cp_2Ti)_3HATN(Ph)_6]$. Although the susceptibility measurements showed the electron transfer to the ligand, the number of transferred electrons cannot be ascertained from the solid-state measurements. In connection with the electrochemical stability of the Ti-free ligand, the transfer of only one electron was assumed in a first assessment. This was also in line with the necessity of a SOMO for explaining the NIR π-π transitions.

Concerning the NIR CT transitions, the MO model discussed above could not explain the disappearance of CT bands for the charge state **+2**. For assessing the contradictions between experimental results and the MO based assignment, DFT calculations were conducted. At this point the limitations of a such simple model became obvious: The arising multiconfigurational problem as evident from DFT calculation needs to be solved by high level quantum calculations. Afterwards, this can be translated back to “partially occupied (single particle) orbitals”. However, in such a situation it becomes impossible to describe an electronic transition as a transition between two orbitals. Rather it should be considered as a transition between two *N*-electron wave functions. Furthermore, our quantum chemical calculations demonstrate, that the MO-diagram of the ligand in the experimental approach was oversimplified and the electronic configuration with the assumption of a transfer of only one electron to the ligand was not justified. In conclusion, the attempt to derive “orbital diagrams” from experimental data alone should be regarded with great caution, since experimental data simply do not allow for an unambiguous assignment of spectral transitions and a subsequent “construction” of “orbital diagrams” due to a lack of atomistic information.

SI-3 Calculated total energies of different species, molecular orbitals and electronic excitations

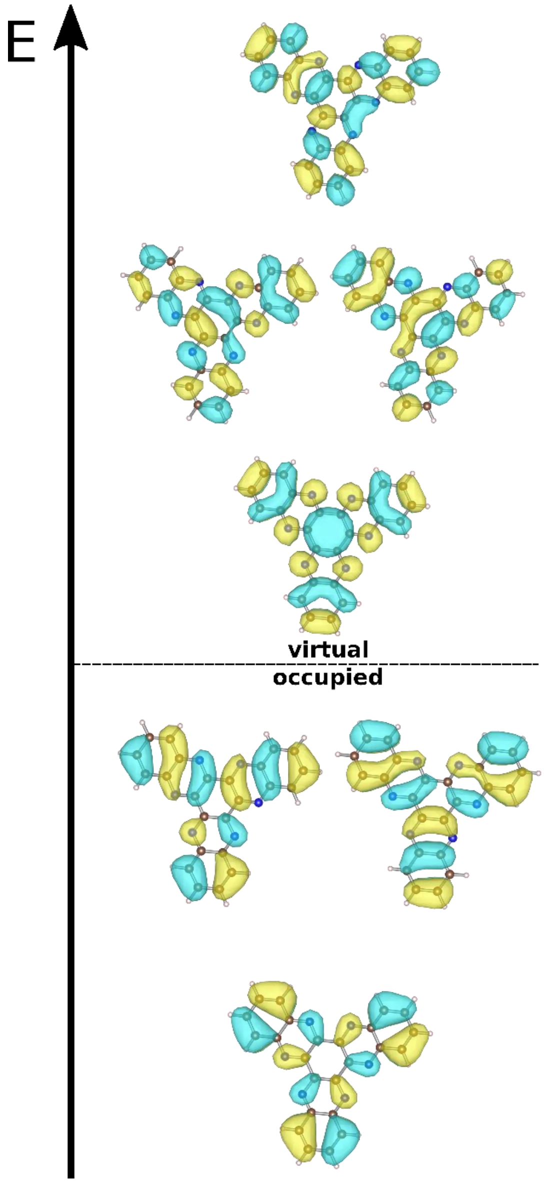

Figure S4. Molecular **o**rbitals of **Ti-free** HATN molecule calculated with PBE0 Def2-SVP.

Table S2. Total energy and spin contamination deviation of different redox species and spin states of $[(Cp_2Ti)_3HATN(Ph)_6]$ with PBE0/RIJCOSX/Def2/J/Def2-SVP. All states were geometry optimized. All charge blocks are referenced to the first multiplicity.

Charge	Multiplicity	Energy [a.u.]	Spin Contamination	ΔE [eV]
0	1	-6338.6436	3.05	0.00
	3	-6338.6428	1.74	0.02
	5	-6338.6423	0.74	0.04
	7	-6338.6428	0.04	0.02
1	2	-6338.4694	2.04	0.00
	4	-6338.4720	0.86	-0.07
	6	-6338.4686	0.04	0.02
2	1	-6338.2041	2.03	0.00
	3	-6338.2042	1.03	0.00
	5	-6338.2037	0.03	0.00
	7	-6338.1405	0.04	1.73

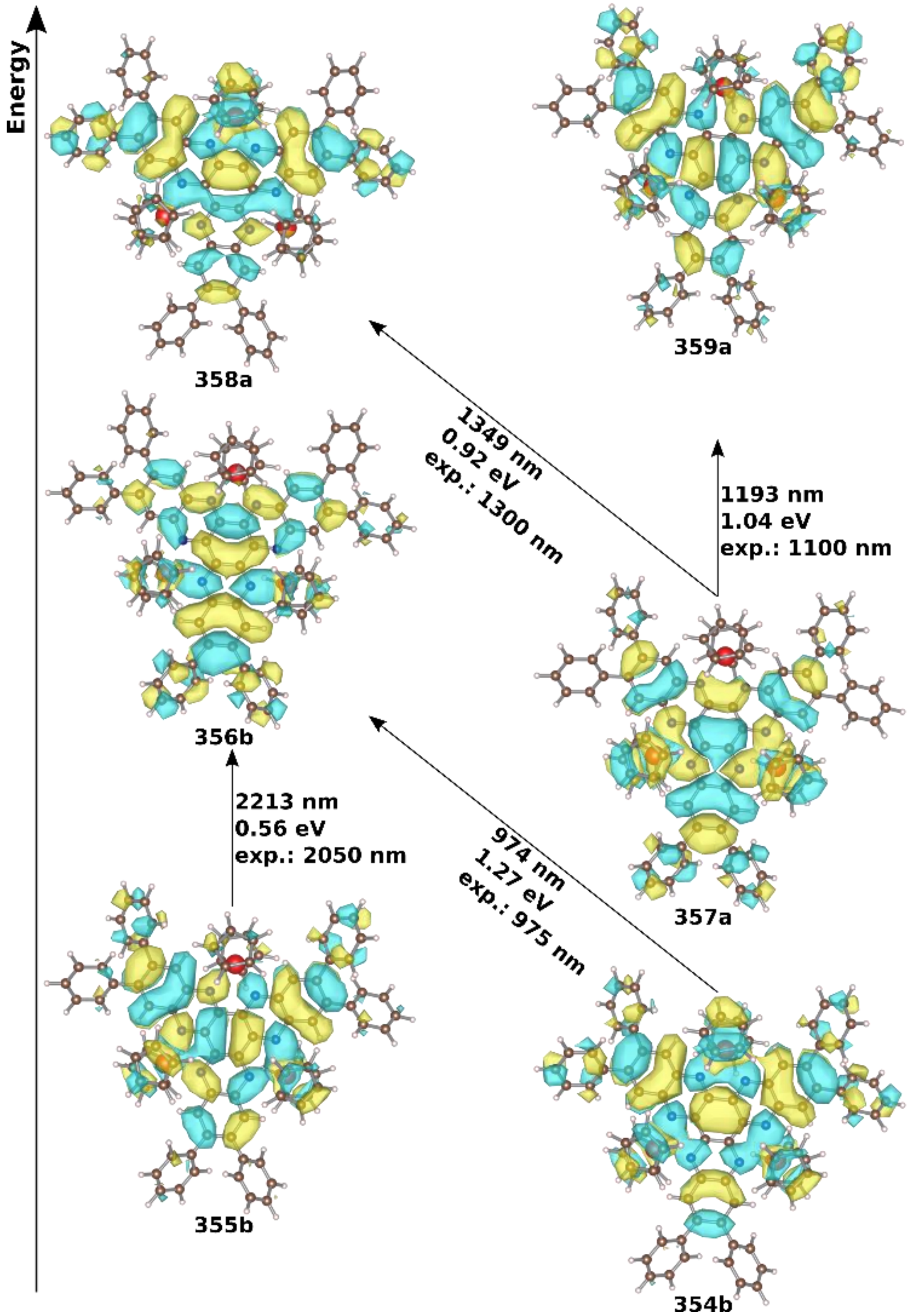

Figure S5. Calculated electronic transitions in the neutral complex [$(Cp_2Ti)_3$HATN$(Ph)_6$] via TD-PBE0. Excitations are compared with experiment (exp. rounded to 25 nm). Molecular orbitals of *S* = 1 and *S* = 2 multiplicity are separated into alpha (a) and beta (b) electrons. Orbitals 354b, 355b and 457a are occupied, orbitals 356b, 358a and 359a are unoccupied. Color scheme of atoms is titanium: red, carbon: brown, nitrogen: blue, hydrogen: white. Orbitals are labelled similar to calculated output.

Table S3. TD-PBE0 excitations and transition dipole moment of $[(Cp_2Ti)_3HATN(Ph)_6]$ in different spin states.

S = 1		*S* = 2	
Wavelength [nm]	T^2 [a.u.^{2}]	Wavelength [nm]	T^2 [a.u.^{2}]
2213.5	0.11	2267.7	0.06
1349.6	0.58	1339.9	0.56
1193.7	3.49	1185.2	3.42
974.0	2.82	986.2	2.86
828.7	0.01	788.2	0.01
764.2	0.01	783.4	0.02
763.2	0.01	686	0.00
660.3	0.00	650.7	0.00
654.8	0.01	642.9	0.01
654.7	0.00	637.6	0.00
630.1	0.00	625.9	0.00
628.6	0.00	624.8	0.00
623.2	0.00	607.5	0.00
599.2	0.00	590.7	0.00
590.4	0.00	588.9	0.06
589.5	0.00	586.9	0.00
583.9	0.00	585.8	0.01
582.7	0.10	575.7	0.03
579.5	0.00	572	0.00
571.4	0.00	565.8	0.00

Table S4. TD-PBE0 excitations and transition dipole moment of $[(Cp_2Ti)_3HATN(Ph)_6]^+$ in different spin states.

S = 1/2		*S* = 3/2	
Wavelength [nm]	T^2 [a.u.^{2}]	Wavelength [nm]	T^2 [a.u.^{2}]
3508.1	0.49	2316.0	0.39
1388.6	5.98	1281.9	0.44
993.5	0.01	1162.1	2.29
976.9	0.00	1059.3	7.78
923.9	0.00	947.7	0.02
684.2	0.01	863.9	0.03
681.8	0.01	810.3	0.01
662.3	0.00	763.5	0.02
657.4	0.00	722.4	0.02
656.2	0.00	671.3	0.00
650.1	0.00	656.1	0.00
643.1	0.02	655.1	0.00
635.8	0.00	652.0	0.01
630.1	0.00	622.7	0.00
601.4	0.01	619.1	0.01
600.0	0.01	612.2	0.00
585.3	0.00	577.4	0.00
564.6	0.00	571.9	0.00
563.5	0.00	566.6	0.00
554.6	0.00	554.4	0.00

Table S5. TD-PBE0 excitations and transition dipole moment of $[(Cp_2Ti)_3HATN(Ph)_6]^{2+}$ in different spin states.

S = 1		*S* = 2	
Wavelength [nm]	T^2 [a.u.^{2}]	Wavelength [nm]	T^2 [a.u.^{2}]
2126.3	7.33	2148.9	7.35
2096.5	7.23	2129.8	7.22
1208.8	0.01	1098.1	0.01
1181.5	0.00	1077.8	0.02
1163.5	0.00	1072.8	0.01
795.5	0.02	1065.1	0.00
784.0	0.02	1052.2	0.05
768.5	0.01	1029.7	0.01
753.6	0.00	693.9	0.00
737.6	0.00	687.4	0.00
724.9	0.00	686.6	0.00
688.5	0.00	664.3	0.58
680.9	0.00	660.2	0.41
680.6	0.00	654.9	0.20
662.0	0.30	632.3	0.02
659.0	0.23	619.7	0.01
654.0	0.23	613.4	0.01
633.9	0.02	611.2	0.08
611.0	0.21	610.4	2.50
610.7	2.78	608.0	2.84

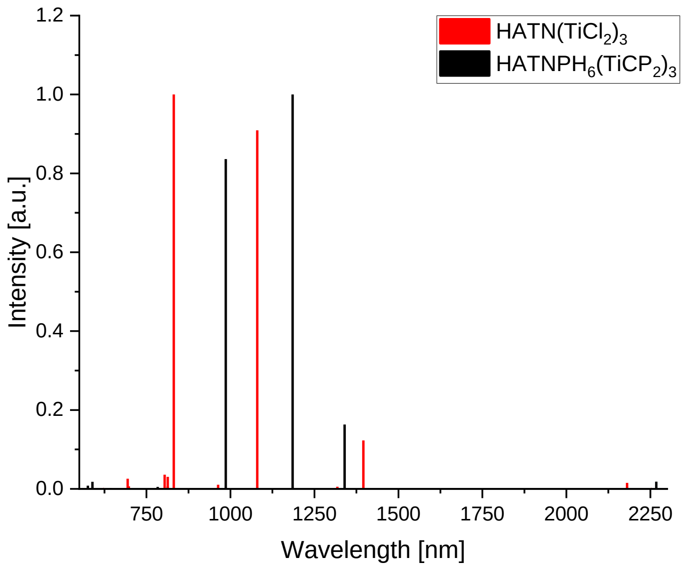

Figure S6. Comparison between TD-PBE0 spectra of simplified model complex (*Q* = 0, *S* = 1) and the synthesized complex (*Q* = 0, *S* = 1).

Table S6. Different spin state total energies of CAS(6,7) and NEVPT2 by including all multiplicities with identical weight in one calculation. All charge blocks are referenced to the lowest multiplicity; Q = 0, Def2-TZVP/RIJCOSX/Def2/JK.

Multiplcity	CAS(6,7) Energy [a.u.]	ΔE [eV]	NEVPT2 Energy [a.u.]	ΔE [eV]
1	-6546.0119	0.00	-6552.97873	0.00
3	-6546.0124	-0.01	-6552.97914	0.00
5	-6546.0126	-0.02	-6552.97935	0.00
7	-6546.0046	0.20	-6552.96225	0.02

Table S7. Orbital occupations of different multiplicities for CAS(6,7); Q = 0, Def2-TZVP/RIJCOSX/Def2/JK.

	Orbital Occupation						
Multiplicity	180	181	182	183	184	185	186
1	1.68	1.00	1.00	1.00	0.99	0.31	0.01
3	1.68	1.00	1.00	1.00	0.99	0.31	0.01
5	1.68	1.00	1.00	1.00	0.99	0.31	0.01
7	1.00	1.00	1.00	1.00	1.00	1.00	0.00

Table S8. Comparison of population for the relevant orbitals between CAS(6,7) model system and a fully symmetrized CAS(12,13) model system. Both calculation were made with Def2-TZVP/RIJCOSX/Def2/JK and Q = 0, S = 1. The first two roots have to be equally weighted for the correct ground state.

CAS(n,m)	Energy [a.u.]	Occupation (relevant orbitals)					
6,7	-6546.0132	1.68	1.00	1.00	1.00	0.99	0.31
12,13	-6546.0671	1.76	1.00	1.00	1.00	1.00	0.25

SI-4 XYZ files of calculated structures

The atom positions in the different calculations are given below.

Table S9. PBE0 RIJCOSX Def2-SVP *S* = 1, *Q* = 0.

Atom	*X* / Å	*Y* / Å	*Z* / Å
Ti	0.3820	4.1318	0.0380
N	1.5606	2.2799	0.1218
N	-1.1113	2.5333	-0.0623
C	1.2028	4.1635	2.3074
C	-0.4574	4.4194	-2.2044
C	-0.1860	3.9135	2.3574
C	0.8141	3.8085	-2.2787
C	-0.8567	5.0615	1.8625
C	1.7754	4.7328	-1.7998
C	0.1211	6.0043	1.4859
C	1.0916	5.9019	-1.4035
C	1.3999	5.4476	1.7538
C	-0.2936	5.7056	-1.6486
C	2.9332	2.1244	0.1519
C	0.8319	1.1495	0.0476
C	-2.4887	2.6371	-0.1011
C	-0.6007	1.2866	-0.0331
H	1.9882	3.4716	2.6059
H	-1.4023	3.9639	-2.4956
H	-0.6623	3.0028	2.7174
H	1.0293	2.8071	-2.6479
H	-1.9350	5.2001	1.8131
H	2.8486	4.5584	-1.7639
H	-0.0803	6.9926	1.0751
H	1.5474	6.8008	-0.9895
H	2.3608	5.9363	1.5924
H	-1.0899	6.4263	-1.4627
C	3.7807	3.2308	0.3041
C	3.5302	0.8290	0.0401
C	1.4191	-0.1308	-0.0259
C	-3.1193	3.8828	-0.2388
C	-3.3138	1.4715	-0.0188
C	-1.4117	0.1351	0.0004
H	3.3156	4.1975	0.4867
C	5.1764	3.1539	0.2711
N	2.7701	-0.3093	-0.0205
C	4.9324	0.7684	-0.0388
C	0.5940	-1.2908	-0.0282
H	-2.4858	4.7545	-0.3952
C	-4.5069	4.0559	-0.2365
N	-2.7709	0.2139	0.0083
C	-4.7049	1.6663	0.0611

C	-0.8147	-1.1581	-0.0347
C	5.7658	1.8847	0.0422
Ti	3.4049	-2.4001	-0.0889
H	5.3816	-0.2051	-0.2249
N	1.2053	-2.5012	-0.0764
C	-5.3222	2.9150	-0.0299
Ti	-3.7647	-1.7058	0.1487
H	-5.3258	0.7898	0.2374
N	-1.6394	-2.2351	0.0020
C	3.2684	-2.1988	2.2793
C	4.6071	-1.9111	1.9138
C	5.1718	-3.0904	1.3827
C	4.1882	-4.1107	1.4307
C	3.0211	-3.5610	2.0010
C	3.0272	-2.3822	-2.4533
C	4.1586	-1.5665	-2.2434
C	5.1874	-2.3689	-1.7076
C	4.7002	-3.6988	-1.6183
C	3.3668	-3.7084	-2.0802
C	0.3856	-3.6005	-0.0720
C	-3.7039	-1.5919	-2.2330
C	-4.9080	-0.9705	-1.8213
C	-5.7186	-1.9504	-1.2131
C	-5.0256	-3.1888	-1.2682
C	-3.7914	-2.9659	-1.9135
C	-3.2033	-1.6756	2.4730
C	-4.2072	-0.6993	2.3163
C	-5.3888	-1.3441	1.8939
C	-5.1208	-2.7361	1.8224
C	-3.7714	-2.9435	2.1826
C	-1.0415	-3.4676	-0.0092
H	2.5546	-1.4920	2.6980
H	5.1027	-0.9504	2.0359
H	6.1871	-3.2093	1.0065
H	4.3236	-5.1429	1.1072
H	2.0857	-4.0897	2.1733
H	2.0637	-2.0489	-2.8353
H	4.2231	-0.4963	-2.4345
H	6.1882	-2.0300	-1.4413
H	5.2632	-4.5613	-1.2663
H	2.7199	-4.5780	-2.1692
C	0.9174	-4.9033	-0.0979
H	-2.8603	-1.0952	-2.7098
H	-5.1576	0.0802	-1.9536
H	-6.7072	-1.7829	-0.7873
H	-5.3893	-4.1470	-0.8988
H	-3.0309	-3.7183	-2.1151

H	-2.1736	-1.4795	2.7681
H	-4.0779	0.3721	2.4641
H	-6.3444	-0.8606	1.6918
H	-5.8384	-3.5084	1.5518
H	-3.2562	-3.8997	2.2514
C	-1.8111	-4.6463	0.0255
H	2.0017	-5.0031	-0.1045
C	0.1438	-6.0623	-0.0644
H	-2.8938	-4.5316	0.0414
C	-1.2726	-5.9326	-0.0103
C	5.9505	4.3891	0.5463
C	5.5723	5.6139	-0.0263
C	7.0263	4.3891	1.4488
C	6.2274	6.7984	0.3048
H	4.7549	5.6362	-0.7513
C	7.6819	5.5717	1.7808
H	7.3399	3.4514	1.9119
C	7.2825	6.7833	1.2163
H	5.9076	7.7360	-0.1573
H	8.5089	5.5469	2.4952
H	7.7944	7.7092	1.4892
C	-5.0461	5.4098	-0.5208
C	-6.0460	5.6000	-1.4883
C	-4.5173	6.5442	0.1146
C	-6.4876	6.8779	-1.8196
H	-6.4695	4.7326	-1.9983
C	-4.9594	7.8246	-0.2156
H	-3.7601	6.4163	0.8926
C	-5.9435	7.9971	-1.1888
H	-7.2591	7.0043	-2.5836
H	-4.5334	8.6902	0.2980
H	-6.2922	8.9974	-1.4578
C	-2.2087	-7.0877	-0.0379
C	-2.0411	-8.1492	-0.9437
C	-3.3270	-7.1221	0.8131
C	-2.9480	-9.2052	-0.9875
H	-1.1873	-8.1465	-1.6245
C	-4.2341	-8.1802	0.7716
H	-3.4824	-6.3143	1.5330
C	-4.0476	-9.2290	-0.1281
H	-2.7904	-10.0172	-1.7025
H	-5.0920	-8.1838	1.4495
H	-4.7551	-10.0619	-0.1587
C	0.8541	-7.3679	-0.0478
C	1.8495	-7.6469	-0.9988
C	0.5811	-8.3387	0.9299
C	2.5365	-8.8603	-0.9860

H	2.0721	-6.9070	-1.7722
C	1.2724	-9.5472	0.9479
H	-0.1834	-8.1386	1.6840
C	2.2466	-9.8174	-0.0144
H	3.2962	-9.0639	-1.7452
H	1.0435	-10.2883	1.7183
H	2.7787	-10.7715	-0.0077
C	7.2135	1.6736	-0.2082
C	7.9205	2.4894	-1.1059
C	7.8874	0.5867	0.3709
C	9.2432	2.2071	-1.4354
H	7.4148	3.3374	-1.5718
C	9.2137	0.3081	0.0476
H	7.3597	-0.0480	1.0874
C	9.8942	1.1121	-0.8657
H	9.7681	2.8451	-2.1510
H	9.7183	-0.5420	0.5134
H	10.9255	0.8795	-1.1421
C	-6.7943	2.9784	0.1571
C	-7.3780	3.9184	1.0224
C	-7.6348	2.0478	-0.4743
C	-8.7502	3.9173	1.2589
H	-6.7423	4.6453	1.5314
C	-9.0095	2.0502	-0.2432
H	-7.2042	1.3189	-1.1661
C	-9.5711	2.9822	0.6286
H	-9.1829	4.6491	1.9460
H	-9.6462	1.3217	-0.7513
H	-10.6469	2.9772	0.8227

Table S10. PBE0 RIJCOSX Def2-SVP, *S* = 2, *Q* = 0.

Atom	*X* / Å	*Y* / Å	*Z* / Å
Ti	0.3818	4.1495	0.0479
N	1.5660	2.2971	0.1031
N	-1.1100	2.5619	-0.0663
C	1.2203	4.1602	2.3158
C	-0.4699	4.4732	-2.1891
C	-0.1661	3.9049	2.3744
C	0.7998	3.8629	-2.2813
C	-0.8450	5.0543	1.8944
C	1.7645	4.7777	-1.7914
C	0.1264	6.0060	1.5225
C	1.0848	5.9419	-1.3734
C	1.4087	5.4512	1.7741
C	-0.3014	5.7504	-1.6132
C	2.9272	2.1375	0.1328
C	0.8254	1.1625	0.0356
C	-2.4774	2.6562	-0.1040
C	-0.5885	1.3035	-0.0447
H	2.0106	3.4680	2.6004
H	-1.4175	4.0232	-2.4804
H	-0.6356	2.9865	2.7236
H	1.0108	2.8650	-2.6621
H	-1.9244	5.1882	1.8521
H	2.8377	4.6012	-1.7632
H	-0.0818	6.9977	1.1234
H	1.5439	6.8338	-0.9480
H	2.3667	5.9451	1.6112
H	-1.0957	6.4690	-1.4113
C	3.7867	3.2420	0.2822
C	3.5251	0.8388	0.0301
C	1.4164	-0.1221	-0.0318
C	-3.1266	3.8989	-0.2272
C	-3.3024	1.4898	-0.0365
C	-1.4025	0.1537	-0.0147
H	3.3278	4.2113	0.4672
C	5.1782	3.1549	0.2476
N	2.7617	-0.2973	-0.0249
C	4.9274	0.7694	-0.0481
C	0.6008	-1.2887	-0.0299
H	-2.5041	4.7804	-0.3724
C	-4.5152	4.0548	-0.2226
N	-2.7468	0.2264	-0.0116
C	-4.6920	1.6641	0.0317
C	-0.8029	-1.1589	-0.0427
C	5.7658	1.8797	0.0232
Ti	3.4041	-2.3980	-0.0801

H	5.3705	-0.2083	-0.2269
N	1.2176	-2.5036	-0.0711
C	-5.3262	2.9066	-0.0389
Ti	-3.7532	-1.6974	0.1090
H	-5.3028	0.7763	0.1854
N	-1.6280	-2.2236	-0.0072
C	3.2653	-2.1831	2.2871
C	4.6042	-1.8955	1.9215
C	5.1710	-3.0770	1.3977
C	4.1885	-4.0983	1.4502
C	3.0202	-3.5472	2.0163
C	3.0334	-2.3951	-2.4461
C	4.1634	-1.5769	-2.2380
C	5.1919	-2.3753	-1.6951
C	4.7054	-3.7047	-1.5984
C	3.3729	-3.7184	-2.0636
C	0.3948	-3.6008	-0.0614
C	-3.6488	-1.5793	-2.2669
C	-4.8590	-0.9541	-1.8780
C	-5.6828	-1.9313	-1.2847
C	-4.9931	-3.1730	-1.3287
C	-3.7477	-2.9543	-1.9519
C	-3.2270	-1.6734	2.4381
C	-4.2254	-0.6925	2.2685
C	-5.4024	-1.3317	1.8269
C	-5.1383	-2.7249	1.7565
C	-3.7964	-2.9386	2.1393
C	-1.0300	-3.4682	-0.0082
H	2.5500	-1.4749	2.7010
H	5.0988	-0.9339	2.0397
H	6.1868	-3.1965	1.0232
H	4.3258	-5.1321	1.1328
H	2.0852	-4.0763	2.1896
H	2.0706	-2.0648	-2.8322
H	4.2272	-0.5081	-2.4367
H	6.1917	-2.0339	-1.4288
H	5.2682	-4.5646	-1.2396
H	2.7263	-4.5888	-2.1472
C	0.9198	-4.9059	-0.0742
H	-2.7972	-1.0859	-2.7330
H	-5.1028	0.0974	-2.0151
H	-6.6777	-1.7600	-0.8754
H	-5.3668	-4.1300	-0.9663
H	-2.9866	-3.7091	-2.1418
H	-2.2026	-1.4829	2.7543
H	-4.0938	0.3776	2.4229
H	-6.3529	-0.8442	1.6110

H	-5.8546	-3.4942	1.4739
H	-3.2861	-3.8965	2.2160
C	-1.8057	-4.6367	0.0250
H	2.0035	-5.0120	-0.0725
C	0.1384	-6.0623	-0.0405
H	-2.8879	-4.5159	0.0316
C	-1.2743	-5.9300	0.0004
C	5.9627	4.3842	0.5211
C	5.5950	5.6120	-0.0515
C	7.0392	4.3744	1.4227
C	6.2617	6.7903	0.2782
H	4.7767	5.6415	-0.7751
C	7.7059	5.5509	1.7538
H	7.3446	3.4340	1.8858
C	7.3175	6.7657	1.1887
H	5.9500	7.7307	-0.1838
H	8.5332	5.5185	2.4675
H	7.8385	7.6870	1.4602
C	-5.0641	5.4098	-0.4865
C	-6.0634	5.6068	-1.4528
C	-4.5408	6.5378	0.1643
C	-6.5090	6.8871	-1.7698
H	-6.4822	4.7439	-1.9740
C	-4.9881	7.8202	-0.1504
H	-3.7820	6.4028	0.9396
C	-5.9703	8.0004	-1.1241
H	-7.2793	7.0200	-2.5338
H	-4.5661	8.6813	0.3738
H	-6.3220	9.0026	-1.3820
C	-2.2162	-7.0798	-0.0298
C	-2.0462	-8.1476	-0.9278
C	-3.3431	-7.1033	0.8101
C	-2.9577	-9.1995	-0.9731
H	-1.1867	-8.1531	-1.6014
C	-4.2555	-8.1568	0.7666
H	-3.5015	-6.2905	1.5238
C	-4.0658	-9.2125	-0.1243
H	-2.7972	-10.0168	-1.6814
H	-5.1198	-8.1513	1.4362
H	-4.7773	-10.0419	-0.1565
C	0.8442	-7.3706	-0.0093
C	1.8434	-7.6611	-0.9524
C	0.5590	-8.3325	0.9732
C	2.5231	-8.8784	-0.9271
H	2.0746	-6.9275	-1.7293
C	1.2433	-9.5448	1.0040
H	-0.2106	-8.1232	1.7196

C	2.2218	-9.8268	0.0496
H	3.2869	-9.0912	-1.6797
H	1.0061	-10.2789	1.7783
H	2.7482	-10.7839	0.0661
C	7.2112	1.6629	-0.2319
C	7.9191	2.4744	-1.1331
C	7.8832	0.5738	0.3458
C	9.2392	2.1858	-1.4674
H	7.4158	3.3241	-1.5985
C	9.2066	0.2881	0.0170
H	7.3560	-0.0571	1.0661
C	9.8875	1.0882	-0.8997
H	9.7641	2.8209	-2.1855
H	9.7092	-0.5636	0.4820
H	10.9166	0.8505	-1.1801
C	-6.7981	2.9476	0.1484
C	-7.3988	3.8704	1.0211
C	-7.6233	2.0041	-0.4853
C	-8.7693	3.8374	1.2650
H	-6.7769	4.6080	1.5320
C	-8.9959	1.9729	-0.2449
H	-7.1816	1.2904	-1.1859
C	-9.5730	2.8857	0.6369
H	-9.2137	4.5557	1.9589
H	-9.6192	1.2330	-0.7532
H	-10.6465	2.8526	0.8401

Table S11. PBE0 RIJCOSX Def2-SVP; *S* = ½, *Q* = 1.

Atom	*X* / Å	*Y* / Å	*Z* / Å
Ti	0.3811	4.1413	0.0415
N	1.5592	2.2552	0.1416
N	-1.1116	2.5165	-0.0506
C	1.1975	4.1800	2.3039
C	-0.4512	4.3967	-2.1995
C	-0.1937	3.9351	2.3506
C	0.8239	3.7890	-2.2594
C	-0.8578	5.0835	1.8479
C	1.7791	4.7238	-1.7874
C	0.1251	6.0193	1.4679
C	1.0883	5.8936	-1.4080
C	1.4012	5.4591	1.7428
C	-0.2955	5.6886	-1.6573
C	2.9331	2.1124	0.1705
C	0.8392	1.1402	0.0669
C	-2.4900	2.6282	-0.0907
C	-0.6089	1.2796	-0.0094
H	1.9788	3.4920	2.6221
H	-1.3914	3.9382	-2.5013
H	-0.6758	3.0352	2.7301
H	1.0471	2.7913	-2.6344
H	-1.9348	5.2327	1.8044
H	2.8544	4.5620	-1.7574
H	-0.0706	7.0089	1.0576
H	1.5387	6.8020	-1.0093
H	2.3636	5.9468	1.5872
H	-1.0941	6.4113	-1.4894
C	3.7711	3.2273	0.3214
C	3.5361	0.8216	0.0565
C	1.4308	-0.1476	-0.0019
C	-3.1118	3.8770	-0.2368
C	-3.3190	1.4681	-0.0031
C	-1.4215	0.1272	0.0259
H	3.3009	4.1912	0.5034
C	5.1628	3.1554	0.2878
N	2.7820	-0.3153	-0.0052
C	4.9397	0.7648	-0.0290
C	0.6096	-1.3050	-0.0086
H	-2.4752	4.7457	-0.3948
C	-4.4964	4.0528	-0.2408
N	-2.7789	0.2091	0.0340
C	-4.7102	1.6643	0.0717
C	-0.8230	-1.1703	0.0012
C	5.7618	1.8842	0.0486
Ti	3.4391	-2.4179	-0.1007

H	5.3931	-0.2054	-0.2205
N	1.1980	-2.5108	-0.0614
C	-5.3196	2.9134	-0.0249
Ti	-3.7939	-1.7137	0.1688
H	-5.3360	0.7915	0.2482
N	-1.6220	-2.2404	0.0370
C	3.3253	-2.2306	2.2571
C	4.6620	-1.9457	1.8803
C	5.2159	-3.1218	1.3328
C	4.2293	-4.1404	1.3844
C	3.0716	-3.5919	1.9724
C	3.0172	-2.3774	-2.4425
C	4.1500	-1.5578	-2.2470
C	5.1908	-2.3587	-1.7357
C	4.7120	-3.6929	-1.6508
C	3.3713	-3.7061	-2.0890
C	0.3877	-3.6072	-0.0583
C	-3.7008	-1.5976	-2.1982
C	-4.9101	-0.9743	-1.8024
C	-5.7283	-1.9516	-1.2025
C	-5.0378	-3.1928	-1.2499
C	-3.7972	-2.9735	-1.8814
C	-3.2384	-1.6819	2.4793
C	-4.2426	-0.7041	2.3205
C	-5.4218	-1.3473	1.8935
C	-5.1553	-2.7411	1.8255
C	-3.8088	-2.9504	2.1913
C	-1.0357	-3.4740	0.0187
H	2.6257	-1.5292	2.7086
H	5.1673	-0.9921	2.0159
H	6.2290	-3.2434	0.9516
H	4.3612	-5.1718	1.0572
H	2.1420	-4.1229	2.1672
H	2.0518	-2.0485	-2.8244
H	4.2094	-0.4879	-2.4416
H	6.1963	-2.0190	-1.4886
H	5.2884	-4.5563	-1.3241
H	2.7344	-4.5820	-2.1868
C	0.9248	-4.9120	-0.0972
H	-2.8598	-1.1063	-2.6857
H	-5.1619	0.0739	-1.9484
H	-6.7240	-1.7842	-0.7938
H	-5.4126	-4.1507	-0.8910
H	-3.0442	-3.7310	-2.0912
H	-2.2155	-1.4883	2.7988
H	-4.1156	0.3657	2.4799
H	-6.3782	-0.8648	1.6931

H	-5.8754	-3.5127	1.5600
H	-3.3018	-3.9093	2.2775
C	-1.8155	-4.6497	0.0527
H	2.0086	-5.0123	-0.1148
C	0.1494	-6.0603	-0.0613
H	-2.8974	-4.5317	0.0777
C	-1.2785	-5.9280	0.0038
C	5.9360	4.3903	0.5647
C	5.5611	5.6125	-0.0146
C	7.0034	4.3867	1.4764
C	6.2156	6.7962	0.3193
H	4.7536	5.6346	-0.7507
C	7.6546	5.5700	1.8139
H	7.3151	3.4497	1.9422
C	7.2606	6.7798	1.2424
H	5.9046	7.7335	-0.1486
H	8.4745	5.5473	2.5359
H	7.7715	7.7054	1.5172
C	-5.0352	5.4042	-0.5362
C	-6.0253	5.5824	-1.5151
C	-4.5136	6.5411	0.0995
C	-6.4646	6.8576	-1.8594
H	-6.4435	4.7120	-2.0246
C	-4.9567	7.8180	-0.2417
H	-3.7676	6.4205	0.8895
C	-5.9301	7.9804	-1.2272
H	-7.2282	6.9787	-2.6317
H	-4.5424	8.6881	0.2730
H	-6.2792	8.9781	-1.5045
C	-2.2094	-7.0868	-0.0221
C	-2.0498	-8.1327	-0.9466
C	-3.3123	-7.1321	0.8476
C	-2.9587	-9.1866	-0.9942
H	-1.2064	-8.1208	-1.6403
C	-4.2173	-8.1916	0.8050
H	-3.4561	-6.3383	1.5857
C	-4.0430	-9.2238	-0.1163
H	-2.8136	-9.9875	-1.7237
H	-5.0615	-8.2120	1.4990
H	-4.7495	-10.0572	-0.1487
C	0.8501	-7.3705	-0.0582
C	1.8334	-7.6480	-1.0217
C	0.5806	-8.3383	0.9226
C	2.5161	-8.8635	-1.0166
H	2.0472	-6.9113	-1.8009
C	1.2730	-9.5461	0.9350
H	-0.1764	-8.1394	1.6846

C	2.2354	-9.8169	-0.0388
H	3.2644	-9.0720	-1.7853
H	1.0529	-10.2865	1.7080
H	2.7655	-10.7719	-0.0372
C	7.2103	1.6881	-0.2046
C	7.9041	2.5148	-1.1021
C	7.8920	0.6044	0.3705
C	9.2272	2.2426	-1.4380
H	7.3919	3.3616	-1.5629
C	9.2200	0.3399	0.0440
H	7.3747	-0.0344	1.0910
C	9.8881	1.1521	-0.8714
H	9.7452	2.8857	-2.1534
H	9.7367	-0.5032	0.5087
H	10.9211	0.9308	-1.1496
C	-6.7910	2.9906	0.1560
C	-7.3635	3.9338	1.0246
C	-7.6372	2.0745	-0.4880
C	-8.7367	3.9488	1.2533
H	-6.7225	4.6506	1.5411
C	-9.0134	2.0973	-0.2679
H	-7.2141	1.3470	-1.1861
C	-9.5655	3.0313	0.6079
H	-9.1646	4.6807	1.9428
H	-9.6588	1.3856	-0.7879
H	-10.6429	3.0437	0.7913

Table S12. PBE0 RIJCOSX Def2-SVP, *S* = 3/2, *Q* = 1.

Atom	*X* / Å	*Y* / Å	*Z* / Å
Ti	0.3832	4.1503	0.0378
N	1.5568	2.2659	0.1436
N	-1.1103	2.5225	-0.0454
C	1.2072	4.2040	2.3004
C	-0.4575	4.4042	-2.2026
C	-0.1828	3.9578	2.3541
C	0.8146	3.7921	-2.2675
C	-0.8502	5.1016	1.8453
C	1.7752	4.7233	-1.7998
C	0.1300	6.0367	1.4562
C	1.0906	5.8972	-1.4206
C	1.4074	5.4797	1.7298
C	-0.2945	5.6970	-1.6632
C	2.9300	2.1192	0.1705
C	0.8356	1.1480	0.0728
C	-2.4878	2.6323	-0.0852
C	-0.6061	1.2857	-0.0108
H	1.9908	3.5189	2.6196
H	-1.4008	3.9491	-2.5000
H	-0.6622	3.0591	2.7398
H	1.0323	2.7921	-2.6395
H	-1.9275	5.2494	1.8054
H	2.8500	4.5568	-1.7729
H	-0.0684	7.0234	1.0402
H	1.5461	6.8045	-1.0254
H	2.3687	5.9675	1.5676
H	-1.0896	6.4232	-1.4938
C	3.7727	3.2311	0.3164
C	3.5301	0.8288	0.0572
C	1.4265	-0.1368	-0.0058
C	-3.1131	3.8804	-0.2280
C	-3.3148	1.4722	0.0002
C	-1.4196	0.1333	0.0312
H	3.3073	4.1981	0.4946
C	5.1648	3.1526	0.2810
N	2.7672	-0.3097	-0.0056
C	4.9310	0.7633	-0.0237
C	0.6022	-1.3045	-0.0050
H	-2.4789	4.7512	-0.3851
C	-4.4979	4.0509	-0.2319
N	-2.7684	0.2119	0.0359
C	-4.7057	1.6614	0.0721
C	-0.8230	-1.1706	-0.0059
C	5.7596	1.8799	0.0489
Ti	3.4320	-2.4154	-0.0859

H	5.3806	-0.2107	-0.2047
N	1.1966	-2.5011	-0.0608
C	-5.3189	2.9090	-0.0202
Ti	-3.7920	-1.7143	0.1591
H	-5.3285	0.7853	0.2423
N	-1.6274	-2.2346	0.0273
C	3.3008	-2.2196	2.2726
C	4.6393	-1.9328	1.9040
C	5.1998	-3.1099	1.3651
C	4.2151	-4.1301	1.4128
C	3.0519	-3.5820	1.9906
C	3.0336	-2.3857	-2.4350
C	4.1643	-1.5656	-2.2307
C	5.1997	-2.3650	-1.7052
C	4.7193	-3.6981	-1.6191
C	3.3833	-3.7126	-2.0715
C	0.3833	-3.6061	-0.0610
C	-3.6984	-1.5889	-2.2100
C	-4.9071	-0.9663	-1.8116
C	-5.7261	-1.9457	-1.2156
C	-5.0360	-3.1866	-1.2672
C	-3.7950	-2.9655	-1.8977
C	-3.2448	-1.6916	2.4744
C	-4.2477	-0.7128	2.3154
C	-5.4258	-1.3540	1.8817
C	-5.1596	-2.7471	1.8089
C	-3.8142	-2.9583	2.1787
C	-1.0376	-3.4740	0.0091
H	2.5966	-1.5183	2.7169
H	5.1420	-0.9779	2.0402
H	6.2158	-3.2306	0.9916
H	4.3516	-5.1626	1.0908
H	2.1219	-4.1143	2.1803
H	2.0725	-2.0587	-2.8287
H	4.2261	-0.4969	-2.4309
H	6.2029	-2.0248	-1.4499
H	5.2917	-4.5604	-1.2823
H	2.7470	-4.5885	-2.1726
C	0.9225	-4.9053	-0.0983
H	-2.8573	-1.0964	-2.6960
H	-5.1590	0.0822	-1.9552
H	-6.7219	-1.7793	-0.8068
H	-5.4112	-4.1457	-0.9119
H	-3.0417	-3.7223	-2.1093
H	-2.2229	-1.4999	2.7985
H	-4.1210	0.3562	2.4810
H	-6.3815	-0.8703	1.6804

H	-5.8789	-3.5176	1.5379
H	-3.3075	-3.9176	2.2625
C	-1.8147	-4.6470	0.0418
H	2.0066	-5.0041	-0.1118
C	0.1478	-6.0591	-0.0665
H	-2.8968	-4.5313	0.0653
C	-1.2750	-5.9288	-0.0045
C	5.9427	4.3867	0.5490
C	5.5698	5.6068	-0.0358
C	7.0129	4.3842	1.4571
C	6.2300	6.7900	0.2888
H	4.7585	5.6280	-0.7678
C	7.6697	5.5669	1.7856
H	7.3229	3.4487	1.9271
C	7.2784	6.7748	1.2080
H	5.9212	7.7257	-0.1836
H	8.4920	5.5454	2.5049
H	7.7936	7.7001	1.4759
C	-5.0410	5.4023	-0.5202
C	-6.0318	5.5819	-1.4981
C	-4.5232	6.5372	0.1219
C	-6.4746	6.8575	-1.8363
H	-6.4485	4.7127	-2.0107
C	-4.9700	7.8144	-0.2132
H	-3.7778	6.4150	0.9121
C	-5.9430	7.9787	-1.1988
H	-7.2379	6.9802	-2.6086
H	-4.5584	8.6833	0.3058
H	-6.2950	8.9767	-1.4713
C	-2.2071	-7.0866	-0.0320
C	-2.0453	-8.1341	-0.9543
C	-3.3131	-7.1295	0.8339
C	-2.9542	-9.1879	-1.0023
H	-1.1994	-8.1238	-1.6449
C	-4.2184	-8.1887	0.7906
H	-3.4597	-6.3337	1.5693
C	-4.0415	-9.2229	-0.1280
H	-2.8077	-9.9900	-1.7303
H	-5.0651	-8.2072	1.4816
H	-4.7481	-10.0562	-0.1607
C	0.8534	-7.3668	-0.0625
C	1.8374	-7.6412	-1.0260
C	0.5880	-8.3350	0.9190
C	2.5243	-8.8544	-1.0208
H	2.0503	-6.9034	-1.8045
C	1.2840	-9.5408	0.9312
H	-0.1686	-8.1376	1.6817

C	2.2466	-9.8090	-0.0432
H	3.2723	-9.0611	-1.7904
H	1.0662	-10.2820	1.7041
H	2.7800	-10.7621	-0.0414
C	7.2071	1.6757	-0.2027
C	7.9042	2.4930	-1.1062
C	7.8848	0.5936	0.3803
C	9.2264	2.2136	-1.4397
H	7.3955	3.3386	-1.5731
C	9.2117	0.3214	0.0555
H	7.3650	-0.0382	1.1052
C	9.8831	1.1246	-0.8654
H	9.7469	2.8494	-2.1599
H	9.7250	-0.5204	0.5262
H	10.9153	0.8973	-1.1420
C	-6.7905	2.9816	0.1611
C	-7.3651	3.9209	1.0325
C	-7.6342	2.0639	-0.4839
C	-8.7379	3.9299	1.2637
H	-6.7261	4.6395	1.5491
C	-9.0100	2.0804	-0.2608
H	-7.2096	1.3395	-1.1844
C	-9.5641	3.0099	0.6185
H	-9.1674	4.6585	1.9557
H	-9.6535	1.3673	-0.7812
H	-10.6410	3.0169	0.8046

Table S13. PBE0 RIJCOSX Def2-SVP; *S* = 1, *Q* = 2.

Atom	*X* / Å	*Y* / Å	*Z* / Å
Ti	0.3851	4.1889	0.0518
N	1.5650	2.2808	0.1416
N	-1.1192	2.5343	-0.0310
C	1.2250	4.2238	2.3013
C	-0.4729	4.4494	-2.1741
C	-0.1661	3.9768	2.3606
C	0.7996	3.8357	-2.2483
C	-0.8356	5.1258	1.8654
C	1.7634	4.7665	-1.7853
C	0.1429	6.0631	1.4779
C	1.0815	5.9390	-1.3980
C	1.4223	5.5029	1.7393
C	-0.3059	5.7396	-1.6321
C	2.9261	2.1351	0.1671
C	0.8340	1.1676	0.0796
C	-2.4846	2.6441	-0.0722
C	-0.6041	1.3046	-0.0005
H	2.0096	3.5422	2.6258
H	-1.4164	4.0007	-2.4806
H	-0.6434	3.0824	2.7593
H	1.0159	2.8432	-2.6413
H	-1.9124	5.2815	1.8442
H	2.8396	4.6068	-1.7779
H	-0.0565	7.0562	1.0781
H	1.5398	6.8483	-1.0107
H	2.3819	5.9957	1.5818
H	-1.0976	6.4692	-1.4612
C	3.7768	3.2475	0.3024
C	3.5236	0.8380	0.0700
C	1.4283	-0.1211	0.0112
C	-3.1156	3.8955	-0.2022
C	-3.3104	1.4773	-0.0090
C	-1.4222	0.1456	0.0320
H	3.3184	4.2182	0.4774
C	5.1632	3.1548	0.2709
N	2.7500	-0.2897	0.0127
C	4.9242	0.7570	-0.0101
C	0.5938	-1.3049	0.0079
H	-2.4854	4.7712	-0.3455
C	-4.4966	4.0527	-0.2212
N	-2.7525	0.2269	0.0287
C	-4.7051	1.6525	0.0464
C	-0.8196	-1.1716	0.0060
C	5.7559	1.8673	0.0437
Ti	3.4428	-2.4250	-0.0805

H	5.3654	-0.2219	-0.1831
N	1.1924	-2.4947	-0.0440
C	-5.3208	2.8950	-0.0290
Ti	-3.8076	-1.7219	0.1397
H	-5.3233	0.7705	0.1997
N	-1.6278	-2.2325	0.0403
C	3.3220	-2.2331	2.2691
C	4.6605	-1.9472	1.8964
C	5.2166	-3.1222	1.3508
C	4.2307	-4.1426	1.3999
C	3.0717	-3.5965	1.9856
C	3.0224	-2.3857	-2.4151
C	4.1527	-1.5605	-2.2172
C	5.1960	-2.3570	-1.7051
C	4.7225	-3.6936	-1.6225
C	3.3831	-3.7129	-2.0634
C	0.3838	-3.6008	-0.0458
C	-3.6698	-1.5966	-2.2177
C	-4.8864	-0.9719	-1.8428
C	-5.7167	-1.9490	-1.2611
C	-5.0271	-3.1919	-1.2983
C	-3.7757	-2.9742	-1.9070
C	-3.2926	-1.6979	2.4525
C	-4.2883	-0.7124	2.2781
C	-5.4636	-1.3463	1.8284
C	-5.2052	-2.7418	1.7629
C	-3.8674	-2.9614	2.1533
C	-1.0409	-3.4693	0.0286
H	2.6277	-1.5392	2.7406
H	5.1707	-0.9986	2.0475
H	6.2330	-3.2454	0.9788
H	4.3681	-5.1756	1.0794
H	2.1465	-4.1318	2.1898
H	2.0625	-2.0658	-2.8179
H	4.2121	-0.4936	-2.4279
H	6.2024	-2.0161	-1.4631
H	5.3050	-4.5557	-1.3026
H	2.7554	-4.5940	-2.1729
C	0.9264	-4.8979	-0.0851
H	-2.8276	-1.1108	-2.7091
H	-5.1415	0.0729	-2.0067
H	-6.7222	-1.7833	-0.8763
H	-5.4140	-4.1505	-0.9543
H	-3.0253	-3.7350	-2.1146
H	-2.2796	-1.5151	2.8085
H	-4.1615	0.3540	2.4598
H	-6.4160	-0.8594	1.6190

H	-5.9282	-3.5084	1.4902
H	-3.3730	-3.9251	2.2567
C	-1.8177	-4.6426	0.0628
H	2.0103	-4.9963	-0.1005
C	0.1535	-6.0514	-0.0502
H	-2.9000	-4.5295	0.0832
C	-1.2758	-5.9221	0.0138
C	5.9550	4.3775	0.5326
C	5.5846	5.6029	-0.0445
C	7.0346	4.3577	1.4306
C	6.2623	6.7764	0.2749
H	4.7694	5.6354	-0.7718
C	7.7028	5.5335	1.7589
H	7.3422	3.4188	1.8949
C	7.3182	6.7459	1.1860
H	5.9637	7.7168	-0.1943
H	8.5300	5.5032	2.4718
H	7.8463	7.6649	1.4498
C	-5.0491	5.3992	-0.4981
C	-6.0470	5.5746	-1.4706
C	-4.5287	6.5331	0.1453
C	-6.4908	6.8500	-1.8062
H	-6.4668	4.7069	-1.9830
C	-4.9830	7.8085	-0.1831
H	-3.7829	6.4119	0.9354
C	-5.9595	7.9703	-1.1665
H	-7.2561	6.9738	-2.5760
H	-4.5776	8.6780	0.3390
H	-6.3157	8.9678	-1.4345
C	-2.2010	-7.0814	-0.0130
C	-2.0300	-8.1301	-0.9334
C	-3.3096	-7.1236	0.8509
C	-2.9371	-9.1848	-0.9830
H	-1.1826	-8.1205	-1.6219
C	-4.2084	-8.1875	0.8092
H	-3.4593	-6.3298	1.5879
C	-4.0249	-9.2214	-0.1091
H	-2.7886	-9.9870	-1.7100
H	-5.0539	-8.2126	1.5013
H	-4.7274	-10.0580	-0.1409
C	0.8559	-7.3576	-0.0447
C	1.8472	-7.6280	-1.0028
C	0.5786	-8.3277	0.9327
C	2.5290	-8.8434	-0.9980
H	2.0652	-6.8904	-1.7803
C	1.2746	-9.5330	0.9467
H	-0.1835	-8.1338	1.6908

C	2.2420	-9.7993	-0.0238
H	3.2779	-9.0529	-1.7655
H	1.0522	-10.2761	1.7161
H	2.7723	-10.7540	-0.0222
C	7.1986	1.6596	-0.2133
C	7.8935	2.4787	-1.1181
C	7.8715	0.5700	0.3633
C	9.2109	2.1904	-1.4613
H	7.3898	3.3300	-1.5798
C	9.1955	0.2944	0.0330
H	7.3555	-0.0586	1.0938
C	9.8642	1.0969	-0.8914
H	9.7311	2.8232	-2.1839
H	9.7104	-0.5471	0.5018
H	10.8947	0.8672	-1.1716
C	-6.7906	2.9624	0.1415
C	-7.3735	3.9029	1.0075
C	-7.6232	2.0335	-0.5038
C	-8.7463	3.8978	1.2367
H	-6.7447	4.6304	1.5239
C	-8.9992	2.0415	-0.2868
H	-7.1925	1.3129	-1.2046
C	-9.5618	2.9686	0.5906
H	-9.1847	4.6230	1.9263
H	-9.6375	1.3263	-0.8100
H	-10.6393	2.9677	0.7726

Table S14. Displacements for model system PBE0 RIJCOSX Def2-TZVP (Q = 0, S = 1).

Atom	X / Å	Y / Å	Z / Å
Ti	-0.0001	3.4757	1.9985
N	-0.0002	1.4213	2.3664
N	-0.0003	2.7814	0.0548
C	-0.0007	0.7164	3.5509
C	-0.0003	0.7031	1.2217
C	0.0003	3.4513	-1.1550
C	-0.0001	1.4199	-0.0048
C	-0.0006	1.3985	4.7814
C	-0.0008	-0.7163	3.5510
C	-0.0002	-0.7031	1.2217
C	0.0004	4.8549	-1.1895
C	0.0002	2.7348	-2.3922
C	0.0002	0.7228	-1.2154
H	-0.0001	2.4916	4.7789
C	-0.0010	0.7027	5.9757
N	0.0001	-1.4212	2.3665
C	-0.0009	-1.3983	4.7814
C	0.0001	-1.4199	-0.0048
H	-0.0001	5.4062	-0.2456
C	0.0005	5.5378	-2.3953
N	0.0000	1.3466	-2.4068
C	0.0003	3.4482	-3.5999
C	0.0002	-0.7228	-1.2154
C	-0.0008	-0.7024	5.9757
Ti	0.0000	-3.4759	1.9985
H	-0.0004	-2.4914	4.7791
N	0.0002	-2.7813	0.0548
C	0.0006	4.8338	-3.6065
Ti	0.0000	0.0000	-3.9911
H	-0.0001	2.8935	-4.5421
N	-0.0002	-1.3466	-2.4069
C	0.0003	-3.4513	-1.1550
C	0.0001	-2.7348	-2.3922
C	0.0002	-4.8548	-1.1894
C	0.0004	-3.4482	-3.5999
H	0.0003	-5.4061	-0.2454
H	0.0006	-2.8936	-4.5421
C	0.0004	-4.8339	-3.6064
H	-0.0006	1.2496	6.9207
H	-0.0004	-1.2493	6.9208
H	0.0001	6.6298	-2.3965
H	0.0003	5.3723	-4.5561
H	0.0007	-5.3725	-4.5560
H	0.0006	-6.6298	-2.3963

C	0.0004	-5.5378	-2.3952
Cl	-1.9417	0.0002	-5.0555
Cl	1.9417	-0.0001	-5.0556
Cl	-1.9429	4.3929	2.5540
Cl	1.9430	4.3927	2.5533
Cl	-1.9432	-4.3929	2.5537
Cl	1.9432	-4.3933	2.5535

Table S15. Displacements for fully symmetrized model system used for CAS(12,13). PBE0 RIJCOSX Def2-TZVP (*Q* = 0, *S* = 1).

Atom	*X* / Å	*Y* / Å	*Z* / Å
Ti	0.0000	3.4758	1.9936
N	0.0000	1.4212	2.3616
N	0.0000	2.7814	0.0500
C	0.0000	0.7163	3.5461
C	0.0000	0.7031	1.2169
C	0.0000	3.4513	-1.1598
C	0.0000	1.4199	-0.0096
C	0.0000	1.3984	4.7766
C	0.0000	-0.7163	3.5461
C	0.0000	-0.7031	1.2169
C	0.0000	4.8548	-1.1943
C	0.0000	2.7348	-2.3970
C	0.0000	0.7228	-1.2203
H	0.0000	2.4915	4.7742
C	0.0000	0.7025	5.9709
N	0.0000	-1.4212	2.3616
C	0.0000	-1.3984	4.7766
C	0.0000	-1.4199	-0.0096
H	0.0000	5.4062	-0.2503
C	0.0000	5.5378	-2.4001
N	0.0000	1.3466	-2.4117
C	0.0000	3.4482	-3.6047
C	0.0000	-0.7228	-1.2203
C	0.0000	-0.7025	5.9709
Ti	0.0000	-3.4758	1.9936
H	0.0000	-2.4915	4.7742
N	0.0000	-2.7814	0.0500
C	0.0000	4.8339	-3.6113
Ti	0.0000	0.0000	-3.9959
H	0.0000	2.8936	-4.5469
N	0.0000	-1.3466	-2.4117
C	0.0000	-3.4513	-1.1598
C	0.0000	-2.7348	-2.3970
C	0.0000	-4.8548	-1.1943
C	0.0000	-3.4482	-3.6047
H	0.0000	-5.4062	-0.2503
H	0.0000	-2.8936	-4.5469
C	0.0000	-4.8339	-3.6113
H	0.0000	1.2494	6.9159
H	0.0000	-1.2494	6.9159
H	0.0000	6.6298	-2.4013
H	0.0000	5.3724	-4.5609
H	0.0000	-5.3724	-4.5609
H	0.0000	-6.6298	-2.4013

C	0.0000	-5.5378	-2.4001
Cl	-1.9417	0.0000	-5.0604
Cl	1.9417	0.0000	-5.0604
Cl	-1.9431	4.3930	2.5488
Cl	1.9431	4.3930	2.5488
Cl	-1.9431	-4.3930	2.5488
Cl	1.9431	-4.3930	2.5488

SI-5 Weighting of configuration state functions for each state involved in electronic excitation and occupation.

Table S16. All CAS(6,7) states involved in NIR electronic excitation with all configuration state functions and their occupation (7 orbitals, (*Q* = 0 , *S* = 1). The specific state is labeled similar to the specific root in the calculation.

State	Configuration state function weighting	Occupation	Energy [a.u.]
0	0.2796	2210100	-6546.0132
	0.2794	2012100	
	0.2015	2111100	
	0.0377	0210120	
	0.0377	0012120	
	0.0328	1210110	
	0.0327	1012110	
	0.0272	0111120	
	0.0237	1111110	
	0.0079	2120100	
	0.0079	2102100	
	0.0071	2021100	
	0.0071	2201100	
	0.0026	1210011	
	0.0025	1012011	
1	0.1910	2120100	-6546.0131
	0.1908	2102100	
	0.1816	2021100	
	0.1815	2201100	
	0.0256	0120120	
	0.0256	0102120	
	0.0244	0021120	
	0.0244	0201120	
	0.0243	2111100	
	0.0222	1120110	
	0.0222	1102110	
	0.0211	1021110	
	0.0211	1201110	
	0.0106	2210100	
	0.0106	2012100	
	0.0038	1111110	
	0.0033	0111120	
12	0.13265	1120110	-6545.9205
	0.13248	1102110	
	0.12657	1021110	
	0.12654	1201110	
	0.07738	1210110	
	0.0773	1012110	

	0.06968	1111110	
	0.02086	2120001	
	0.02082	2102001	
	0.0199	2021001	
	0.01989	2201001	
	0.0146	2120100	
	0.01458	2102100	
	0.01394	2021100	
	0.01393	2201100	
	0.01217	2210001	
	0.01215	2012001	
	0.01097	2111001	
	0.00853	2210100	
	0.00852	2012100	
	0.00721	2111100	
	0.0045	1120011	
	0.00449	1102011	
	0.00429	1021011	
	0.00429	1201011	
	0.00374	0120120	
	0.00373	0102120	
	0.00357	0021120	
	0.00357	0201120	
	0.00263	1210011	
	0.00262	1012011	
18	0.12885	1120110	-6545.8660
	0.12868	1102110	
	0.12293	1021110	
	0.1229	1201110	
	0.0763	1111110	
	0.06998	1210110	
	0.0699	1012110	
	0.0174	0120120	
	0.01737	0102120	
	0.0166	0021120	
	0.0166	0201120	
	0.01177	0120201	
	0.01175	0102201	
	0.01123	0021201	
	0.01122	0201201	
	0.01045	0111120	
	0.00942	0210120	
	0.00941	0012120	
	0.00871	1120011	
	0.00869	1102011	
	0.00831	1021011	
	0.0083	1201011	

	0.00702	0111201	
	0.00639	0210201	
	0.00638	0012201	
	0.00538	0120021	
	0.00537	0102021	
	0.00528	1111011	
	0.00513	0021021	
	0.00513	0201021	
	0.00474	1210011	
	0.00473	1012011	
	0.00343	1111020	
	0.00324	0111021	
	0.00322	1120020	
	0.00321	1102020	
	0.00307	1021020	
	0.00307	1201020	
	0.00292	0210021	
	0.00292	0012021	
27	0.10338	0210120	-6545.8185
	0.10327	0012120	
	0.08669	0120120	
	0.08657	0102120	
	0.08291	0021120	
	0.0829	0201120	
	0.06153	0111120	
	0.03671	1210110	
	0.03667	1012110	
	0.03068	1120110	
	0.03064	1102110	
	0.02934	1021110	
	0.02933	1201110	
	0.02191	1111110	
	0.01524	1210011	
	0.01523	1012011	
	0.01271	1120011	
	0.01269	1102011	
	0.01216	1021011	
	0.01215	1201011	
	0.0121	2210100	
	0.01208	2012100	
	0.01016	2120100	
	0.01015	2102100	
	0.00972	2021100	
	0.00972	2201100	
	0.00904	1111011	
	0.00721	2111100	
30	0.10848	2120001	-6545.7919

0.10832	2102001
0.1036	2021001
0.10357	2201001
0.08631	2210001
0.08621	2012001
0.06256	2111001
0.01692	1120110
0.0169	1102110
0.01617	1021110
0.01616	1201110
0.01424	0120201
0.01421	0102201
0.0136	0021201
0.01359	0201201
0.01345	1210110
0.01344	1012110
0.01132	0210201
0.01131	0012201
0.0099	1111110
0.00823	0111201
0.00774	0120021
0.00773	0102021
0.00739	0021021
0.00739	0201021
0.00647	0120120
0.00646	0102120
0.00618	0021120
0.00618	0201120
0.00616	0210021
0.00615	0012021
0.00545	2120100
0.00544	2102100
0.00521	2201100
0.00521	2021100
0.00518	0210120
0.00517	0012120
0.00454	1120011
0.00453	1102011
0.00448	0111021
0.00434	1021011
0.00433	2210100
0.00433	2012100
0.00433	1201011
0.00361	0111120
0.00355	1210011
0.00355	1012011
0.00316	1111011

0.00316	2111100

Table S17. Oscillator strength and transition dipole moments of electronic excitations within CAS(6,7)/NEVPT2 (Q=0, S=1).

Transition (state0→stateX)	Fosc	T^2 [a.u.]^2
0→12	0.00388	2.36
0→18	0.05615	19.86
0→27	0.04477	9.21
0→30	0.09020	14.35

References

[1] R. Holze, *Organometallics* **2014**, *33*, 5033–5042.

[2] V. Balzani, G. Bergamini, S. Campagna, F. Puntoriero in *Topics in Current Chemistry*, *Vol. 280* (Eds.: V. Balzani, S. Campagna), Springer Berlin Heidelberg, Berlin, Heidelberg, **2007**.

[3] P. Sander, A. Markovic, M. Schmidtmann, O. Janka, G. Wittstock, R. Beckhaus, *Inorg. Chem.* **2018**, *57*, 11165–11174.

[4] P. Peljo, H. H. Girault, *Energy Environ. Sci.* **2018**, *11*, 2306–2309.

[5] A. M. Bond, E. A. McLennan, R. S. Stojanovic, F. G. Thomas, *Anal. Chem.* **1987**, *59*, 2853–2860.

[6] S. Trasatti, *Pure Appl. Chem.* **1986**, *57*, 955–966.

[7] a) H. Reiss, *J. Phys. Chem.* **1985**, *89*, 3783–3791; b) P. W. Atkins, T. L. Overton, J. P. Rourke, M. T. Weller, F. A. Armstrong, *Shriver and Atkins' Inorganic Chemistry,* 5th ed., Oxford University Press, Oxford, **2010**;
